# π-extended anthracenes as sensitive probes for mechanical stress†
†Electronic supplementary information (ESI) available: Detailed synthetic, spectroscopic, kinetic and mechanochemical procedures. See DOI: 10.1039/c5sc03297k



**DOI:** 10.1039/c5sc03297k

**Published:** 2015-10-07

**Authors:** R. Göstl, R. P. Sijbesma

**Affiliations:** a Laboratory of Supramolecular Polymer Chemistry , Department of Chemical Engineering and Chemistry , Eindhoven University of Technology , P.O. Box 513 , 5600 MB Eindhoven , The Netherlands . Email: r.gostl@tue.nl ; Email: r.p.sijbesma@tue.nl

## Abstract

Smart molecular systems having the ability to report on mechanical strain or failure in polymers *via* alteration of their optical properties are of great interest in materials science. However, only limited attention has been devoted to targeted chromophore engineering to fine-tune their physicochemical properties. Here, we describe the synthesis of π-extended anthracenes that can be released from their respective maleimide Diels–Alder adducts through the application of mechanical stress in solution and in the solid state. We demonstrate the improvement of fluorescence quantum yield as well as the tuning of excitation and emission wavelengths while retaining their excellent mechanochemical properties laying the foundation for a new series of mechanophores whose spectral characteristics can be modularly adjusted.

## Abstract 2

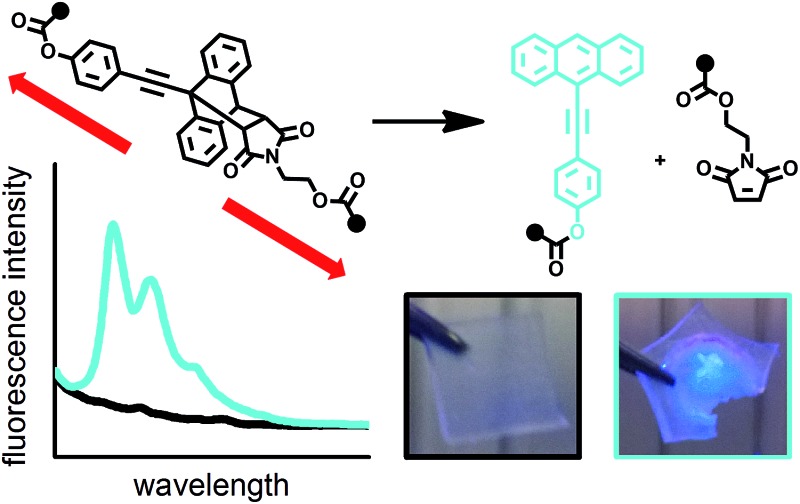
Diels–Alder adducts of π-extended anthracenes have been synthesised, employed as mechanophores in polymeric materials and show unprecedented detection sensitivity for mechanical stress.

## Introduction

**Fig. 1:**
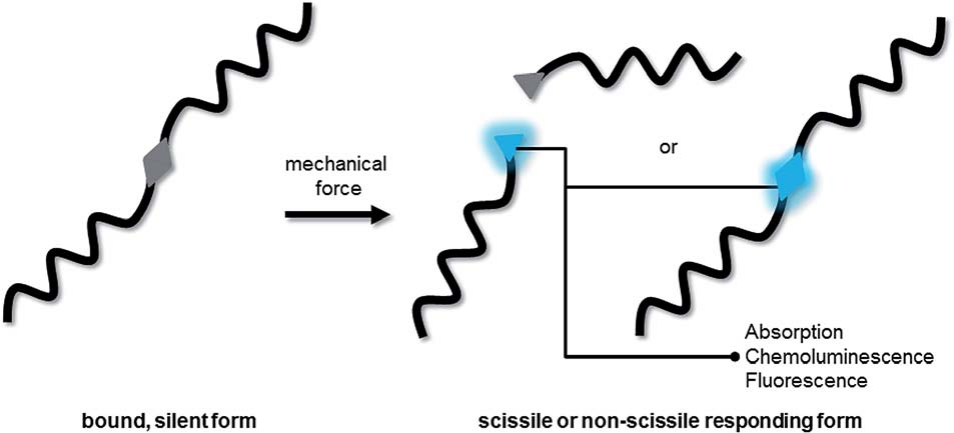
Schematic depiction of a scissile or non-scissile moiety incorporated into a polymer altering its optical properties upon the application of mechanical force.

Molecular systems that report mechanical strain or failure are highly sought-after in the field of smart materials. A common and straight-forward approach for the implementation of this function into polymeric materials is to incorporate molecular moieties (mechanophores) in the polymer backbone that alter their physicochemical properties upon exposure to mechanical stress.^1–5^ One of the most simple modes of detection is the change in optical properties, as this method is not only non-invasive and spatiotemporally well resolved but in principle also detectable with the naked eye (Fig. 1). Moore, White, Sottos and co-workers tackled this challenge and established spiropyran as now widely employed mechanochromic probe.^1,6,7^ Other mechanochromic moieties have been successfully activated in polymers, including azobenzene,^8^ diaryl-bibenzofuranone^9^ and the coumarin dimer.^10^ Also, the reorientation of chromophores in polymer blends has been exploited to modulate materials' absorption spectra.^11–13^ However, measurement of a change in absorption is generally less sensitive than measuring a change in emission. To enable higher detection sensitivity, we established the chemoluminescent dioxetane motif as a highly sensitive probe to observe material failure.^14–18^ Intrinsically however, the nature of chemoluminescence does not allow observation of the material's failure independent of time. Consequently, mechanofluorochromism – the activation or alteration of fluorescence *via* mechanical stress – could qualify as the most promising pathway to achieve persistent ultra-sensitive detection. Indeed, the widely employed spiropyran motif's merocyanine form has also been successfully employed as fluorometric probe,^19–25^ even though its low fluorescence quantum yield of *φ*_f_ < 0.02 does not render it an ideal candidate for this purpose.^26,27^ The mechanically induced retro Diels–Alder (DA) reaction releasing anthracene as fluorophore^28–31^ (*φ*_f_ = 0.27)^32^ seems much more promising. However, anthracene's comparably low *φ*_f_, its absorption spectrum lying exclusively in the UV region as well as its fluorescence's high susceptibility to oxygen quenching still leave plenty of room for improvement.

**Chart 1:**
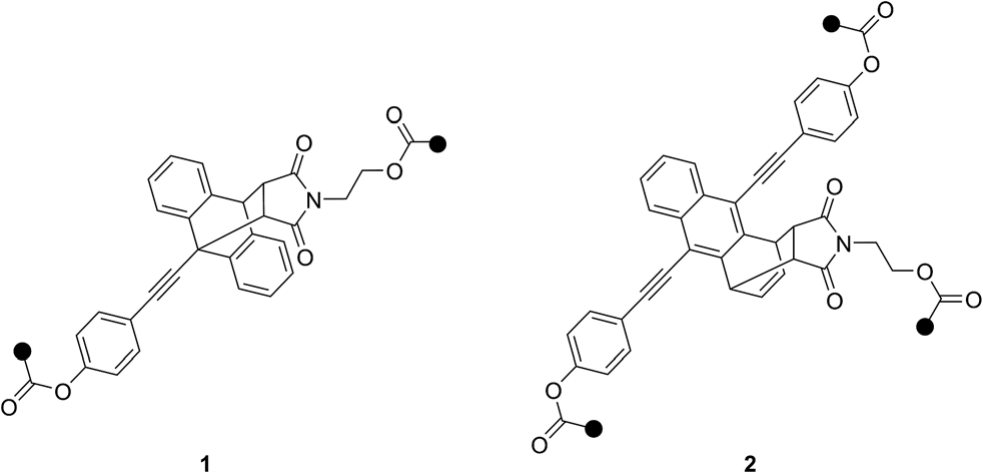
Diels–Alder adduct motifs **1** and **2** of π-extended anthracenes studied as mechanophores.

Overcoming these molecular systems' limitations, we here report on the design and synthesis of a novel 9-π-extended anthracene with outstanding fluorescence quantum yield, low oxygen sensitivity and bathochromically shifted absorption- and emission-spectrum. We show the incorporation of its corresponding 9,10-maleimide DA adduct **1** into linear poly(methyl acrylate) (PMA) as well as a crosslinked poly(hexyl methacrylate) (PHMA) network (Chart 1). The mechanically induced retro DA reaction is proven to work in solution *via* sonication as well as in the solid state and is accompanied by detailed spectroscopic and kinetic analyses. Moreover and additionally to the traditionally observed 9,10-adduct, we here unravel the spectroscopic and mechanochemical peculiarities of the rarely reported 1,4-adduct **2** formed from a 9,10-π-extended anthracene and maleimide.

## Results and discussion

### Synthesis

**Scheme 1:**
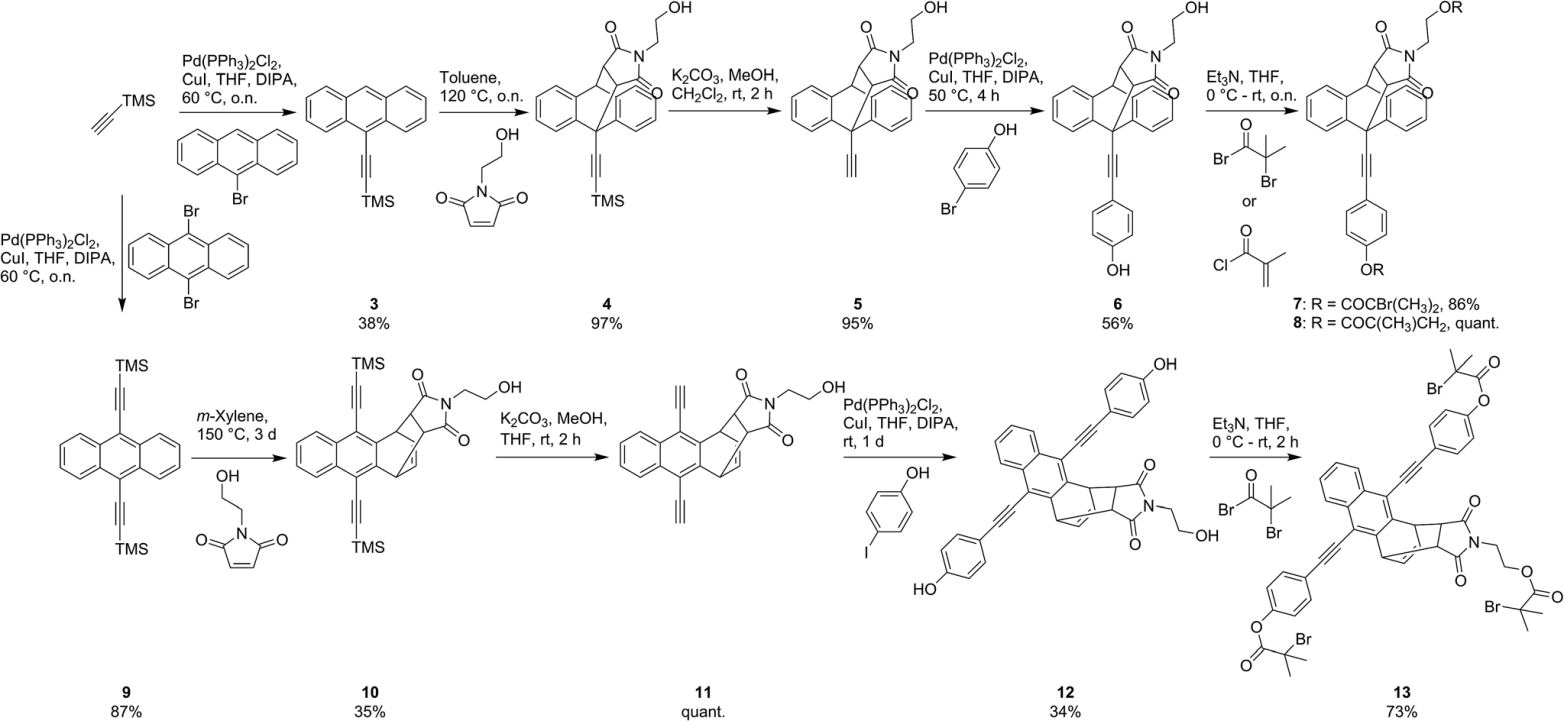
Synthesis of initiator or crosslinker DA adducts **7**, **8** and **13**.

The pathway towards functionalized mechanophores **7** and **8** started with the Pd-catalyzed Sonogashira-type cross-coupling of TMS-acetylene to commercial 9-bromoanthracene yielding ethynylated derivative **3** in moderate yields (Scheme 1). Subsequent Diels–Alder reaction of **3** with *N*-(2-hydroxyethyl)maleimide (NHEM) – prepared according to Haddleton and co-workers^33^ – gave 9,10-adduct **4** in quantitative yield. Basic deprotection of **4** to terminal alkyne **5** and subsequent Sonogashira-type cross-coupling with 4-bromophenol yielded dialcohol **6**. This precursor was then either esterified with α-bromoisobutyryl bromide to yield bisinitiator **7** determined for SET-LRP or with methacryloyl chloride to give crosslinker **8** for application in bulk free radical polymerization.

**Chart 2:**
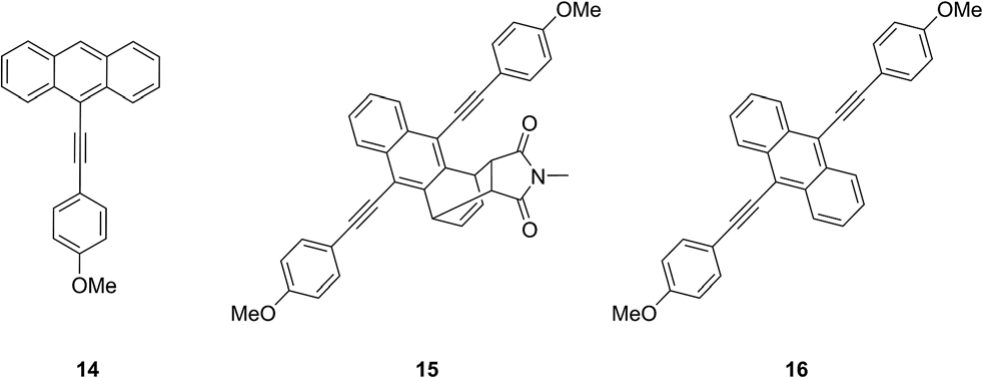
Reference compounds **14–16**.

Analogously, synthesis towards tri-initiator **13** started with the double Sonogashira-type cross-coupling of TMS-acetylene to commercial 9,10-dibromoanthracene. Interestingly, the Diels–Alder reaction of product **9** with NHEM did not result in the commonly expected 9,10-adduct but to our surprise yielded 1,4-adduct **10** almost exclusively. To the best of our knowledge this reactivity pattern is very uncommon and can be attributed to excessive steric crowding of the anthracene's 9- and 10-positions rendering these locations inaccessible for the dienophile.^34^ Deprotection of **10** yields light and temperature sensitive terminal alkyne **11** which was reacted with 4-iodophenol in a mild Sonogashira-type cross-coupling giving trialcohol **12**. Subsequent esterification with α-bromoisobutyryl bromide yielded trifunctional initiator **13**. Additionally, for the determination of molar absorptivities and fluorescence quantum yields, reference compounds **14–16** were synthesized (Chart 2, see ESI† for details).

Attachment of PMA chains to initiators **7** and **13** was carried out by employing SET-LRP conditions established by Haddleton and co-workers^35^ as well as Percec and co-workers^36^ to yield two-armed **PMA-1** (*M*_n_ = 60 kD, *M*_w_/*M*_n_ = 1.12) and three-armed **PMA-2** (*M*_n_ = 110 kD, *M*_w_/*M*_n_ = 1.17) respectively.

Incorporation of crosslinker **8** into a network was performed by dibenzoyl peroxide initiated bulk free radical polymerization of HMA yielding network **PHMA-1**. A reference network blend containing non-covalently bound **7** was prepared in a similar fashion resulting in **PHMA-7**. The experimental details are summarized in the ESI.†

### 9,10-Diels–Alder adduct of 9-π-extended anthracene

**Scheme 2:**
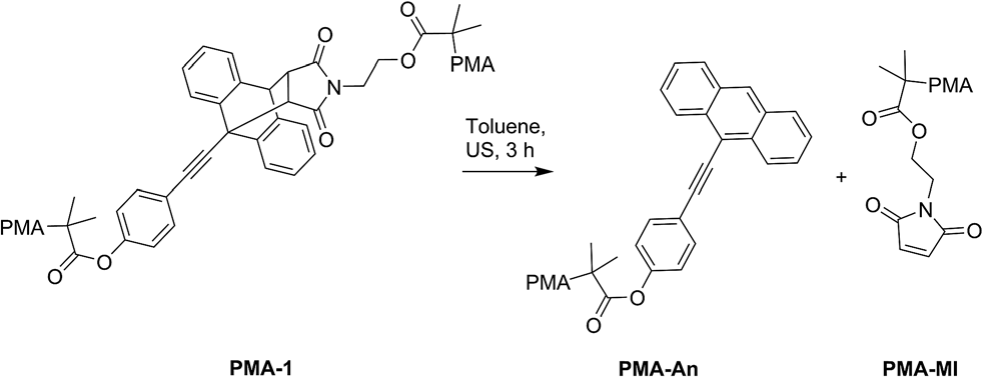
Scission of **PMA-1** applying ultrasound (US).

**Fig. 2:**
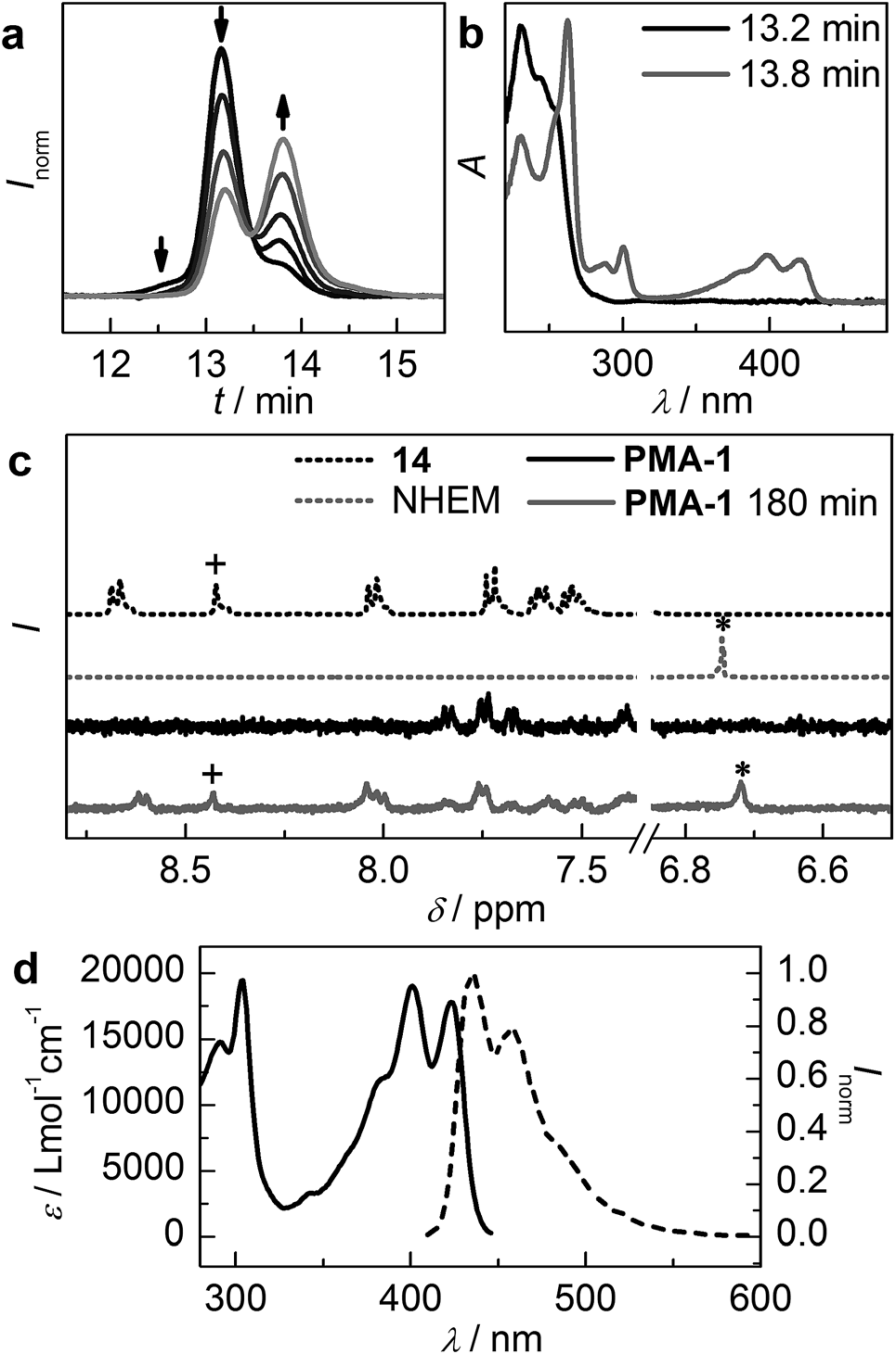
Sonochemical scission of **PMA-1** monitored *via* (a) the RI-trace of GPC chromatograms at different times of irradiation with ultrasound, (b) the UV/vis spectra of the initial high molecular weight peak and the newly formed low molecular weight peak after 180 min of irradiation with ultrasound as extracted from the PDA detector of the GPC and (c) sections of the ^1^H-NMR spectra of reference compound **14**, NHEM, untreated **PMA-1** and **PMA-1** after 180 min of sonication (+anthracene's 10-proton, *vinylic maleimide proton). (d) UV/vis absorption and normalized fluorescence spectrum of reference compound **14** (*λ*_exc_ = 372 nm).

Proof of principle for **PMA-1**'s ability to undergo mechanochemically induced scission was provided by employing irradiation with ultrasound. As the mechanophore is located in the centre of the polymer, the scission is expected to take place *via* the retro DA reaction producing a maleimide (**PMA-MI**) and a 9-phenylethynylanthracene-terminated (**PMA-An**) fragment (Scheme 2). The irradiation with ultrasound was observed by gel permeation chromatography (GPC) through the refractive index (RI) detector trace (Fig. 2a). It can be clearly seen that the high molecular weight peak (13.2 min) is depleted and a new peak corresponding to half of the initial molecular weight (13.8 min) is formed. Extraction of the UV/vis spectra of both peaks from the GPC's photodiode array (PDA) detector clearly shows that a chromophore absorbing in the visible region is formed during the sonication process (Fig. 2b). Comparison with the absorption spectrum of reference compound **14** confirms that the formed chromophore moiety is indeed the anthracene (Fig. 2d). Moreover, ^1^H-NMR measurements unambiguously prove the formation of free maleimide and anthracene moieties (Fig. 2c). Comparison with reference compound **14** clearly demonstrates the regeneration of the characteristic signal of anthracene's proton in 10-position (+) and the reformation of the maleimide's vinylic protons (*).

Kinetic analysis of the scission process was carried out by assuming a unimolecular reaction following a first-order mechanism. The normalized peak heights of the GPC RI-traces were employed to estimate the individual polymer fragments' relative concentrations^28,37^ and subsequently the scission constant *k* was determined to (4.6 ± 0.1) × 10^–3^ min^–1^.

An alternate method for the determination of *k* is to follow the mechanical scission by UV/vis spectroscopy. As the molar absorptivity *ε* of **PMA-An** is known from reference **14**, *k* can be determined to (4.6 ± 0.2) × 10^–3^ min^–1^. These values are identical within the experimental error and are comparable to those determined for regular anthracenes by Boydston and co-workers, rendering **1** an excellent mechanophore.^28^

Most importantly however, the spectral characteristics of the 9-π-extended anthracene **14** render motif **1** a superior force-sensitive probe. Firstly, **14** absorbs in the visible region of the spectrum enabling excitation of the fluorophore with a visible light source (Fig. 2d). This avoids the generally unwanted irradiation of the material with destructive UV light. Moreover and in contrast to unsubstituted anthracene, the complete emission spectrum lies in the visible range of the spectrum facilitating detection with the naked eye. Above all however, **14**'s fluorescence quantum yield was determined to *φ*_f_ = 0.72 which is almost two orders of magnitude higher than the widely employed merocyanine and a considerable improvement over bare anthracene. In air-saturated solution **14** still reaches *φ*_f_ = 0.62 rendering the fluorescence only mildly susceptible to ^3^O_2_ quenching. Interestingly, this unusual behaviour and the low reactivity towards ^1^O_2_ is known from literature for ethynylated anthracenes and is attributed to their comparably low tendency to undergo intersystem crossing and short triplet lifetimes as compared to anthracene.^38,39^

**Fig. 3:**
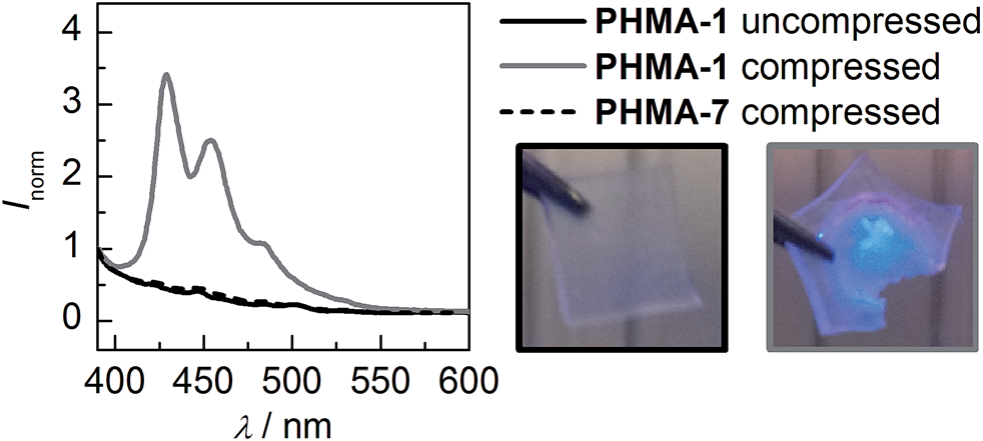
Normalized solid state fluorescence spectra of uncompressed and compressed 6 × 6 × 0.5 mm **PHMA-1** as well as compressed reference **PHMA-7** samples (*λ*_exc_ = 372 nm). The boxes show corresponding pictures of the samples while irradiated with a UV hand lamp (*λ*_exc_ = 365 nm).

These results encouraged us to advance our system to the solid state by incorporating motif **1** into a crosslinked PHMA network. Employing a pellet press, a weight-equivalent of 10 t was exerted on a 6 × 6 × 0.5 mm sample of **PHMA-1**. The change in fluorescence is immediately visible and solid state fluorescence spectroscopy confirms the generation of reference **14**'s typical emission spectrum (Fig. 3). Due to the system's irreversibility at ambient conditions, the signal intensity remains independent of time. Moreover, we showed that simple blending of **7** into a PHMA network (**PHMA-7**) with subsequent compression does not initiate the retro DA reaction confirming the mechanochemical origin of the observed process.

### 1,4-Diels–Alder adduct of 9,10-π-extended anthracene

**Fig. 4:**
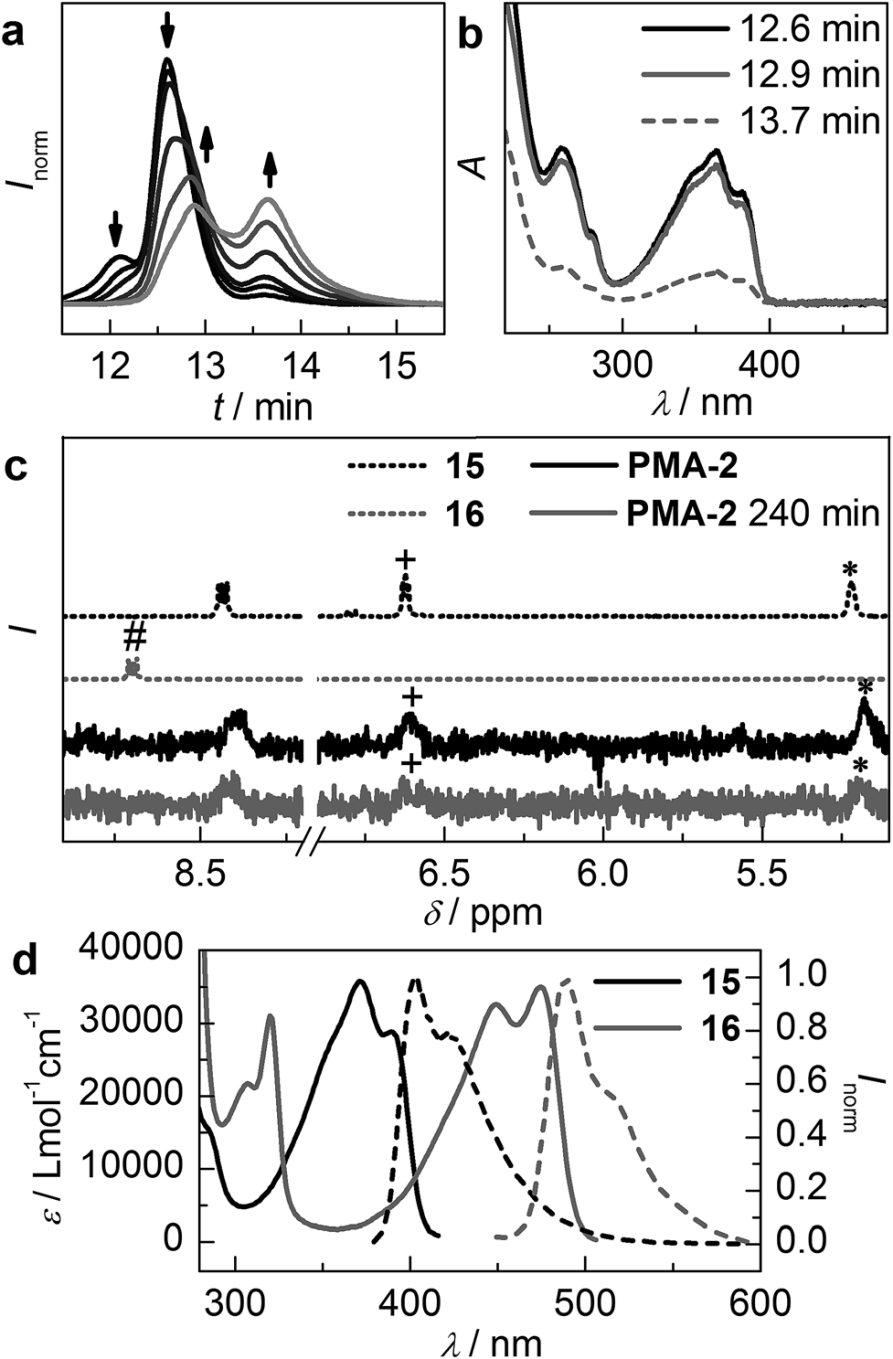
Sonochemical scission of **PMA-2** monitored *via* (a) the RI-trace of GPC chromatograms at different times of irradiation with ultrasound, (b) the UV/vis spectra of the initial high molecular weight peak and the newly formed low molecular weight peaks after 240 min of irradiation with ultrasound as extracted from the PDA detector of the GPC and (c) sections of the ^1^H-NMR spectra of reference compounds **15**, **16**, untreated **PMA-2** and **PMA-2** after 240 min of sonication (#anthracene's 1-, 4-, 5- and 8-protons, +DA adduct's vinylic proton and *DA adduct's allylic proton). (d) UV/vis absorption and normalized fluorescence spectra of reference compounds **15** and **16** (*λ*_exc_ = 372 nm).

These satisfying results led us to investigate the attachment of phenylethynyl-moieties to anthracene's 9- and 10-positions, as this should result in even more bathochromically shifted absorption and emission spectra. Moreover, fluorescence quantum yields of 9,10-π-extended anthracenes were proven to almost approach unity.^38,40^ Additionally, the unconventional 1,4-DA adduct would enable not only turn-on fluorescence upon scission but fluorescence switching from blue to green, as the anthracene's π-system remains only partially disrupted in the adduct form (compare Fig. 4d).

For the sonochemical proof of concept experiment we employed three-armed **PMA-2**, which was supposed to undergo the retro DA reaction similar to **PMA-1**, as it was shown by Boydston and co-workers that an additional third arm does in principle not alter the anthracene DA adduct's scission rate.^28^ Indeed, GPC chromatogram RI traces recorded during the course of **PMA-2**'s irradiation with ultrasound reveal the expected decrease of the initial high molecular weight peak (12.6 min) and the formation of two new fragments, one at 2/3 (12.9 min) and one at 1/3 (13.7 min) of the initial molecular weight indicating scission of one polymer arm (Fig. 4a). The shoulder at 12.1 min of the initial, non-irradiated trace is attributed to recombination-chain termination during the SET-LRP synthesis. However, inspection of the UV/vis spectra extracted from the GPC's PDA detector reveals that the chromophore remains unaltered in the 2/3 (12.9 min) fragment and appears to be only in minor presence in the 1/3 (13.7 min) fragment (Fig. 4b). Comparison of the UV/vis traces with those of reference compounds **15** and **16** clearly indicates that no scission *via* the retro DA reaction has taken place (Fig. 4d). ^1^H-NMR measurements unambiguously confirm this assumption. The DA adduct's characteristic allylic (*) and vinylic (+) proton signals can be found before and after the irradiation with ultrasound (Fig. 4c). Furthermore, the typical downfield shifted signal of the free anthracene's protons (#) cannot be retrieved after sonication.

This unexpected result can be rationalized in two different ways. On the one hand it is possible that the 1,4-DA adduct of this anthracene derivative is not mechanochemically active. This could result from a high activation barrier for the retro DA reaction and/or a high thermodynamic stability of the adduct. On the other hand it is also likely that the mechanical force vector is not aligned with the reaction coordinate. Just as Makarov, Bielawski and co-workers predicted and observed for classical 9,10-adducts,^31^ it could be necessary to attach the force transmitting PMA chains directly to the carbon-atom involved in the pericyclic reaction.

## Conclusions

The π-extension of anthracenes and their incorporation into mechanochemically active DA adducts was shown to be an excellent method to substantially improve these stress-reporting optical probes as compared to bare anthracene. By thoughtful introduction of substituents we were able to synthesize a mechanophore that releases an anthracene with a fluorescence quantum yield as high as 0.72 through the application of mechanical stress in solution as well as in the solid state. This value is almost 2 orders of magnitude higher than the currently widely employed spiropyran-merocyanine system and moreover neither prone to thermal reversibility nor to excessive oxygen quenching. We here laid the foundation for a new series of mechanofluorochromic probes whose physicochemical properties can be fine-tuned to absorb and emit light at desirable wavelengths with high efficiency combining the strengths of targeted chromophore engineering with polymer mechanochemistry.
