# Supplementary material for: π-extended anthracenes as sensitive probes for mechanical stress
Source: Chem Sci. 2015 Oct 7;7(1):370–5. doi: 10.1039/c5sc03297k (PMC5518546; doi:10.1039/c5sc03297k)
Supplement: Supplementary file 1 [file SC-007-C5SC03297K-s001.pdf]

Electronic Supplementary Material (ESI) for Chemical Science.
This journal is © The Royal Society of Chemistry 2015

Electronic Supplementary Information

for

Π-extended anthracenes as sensitive probes for mechanical stress

R. Göstl* and R. P. Sijbesma*

Technische Universiteit Eindhoven, Molecular Science and Technology, group Supramolecular Polymer Chemistry, P.O. Box 513, 5600 MB Eindhoven

Contents

1. Materials and Methods S3

1.1. Analytical Instrumentation S3

1.2. Chemicals and Solvents S4

2. Synthetic Procedures and Characterization Data S5

2.1. Target Compounds 7, 8, and 13 .... S5

2.1.1. (Anthracen-9-ylethynyl)trimethylsilane 3 .... S5

2.1.2. (Anthracen-9-ylethynyl)trimethylsilane *N*-(2-hydroxyethyl)maleimide 9,10-Diels-Alder adduct 4 .... S6

2.1.3. 9-Ethynylanthracene *N*-(2-hydroxyethyl)maleimide 9,10-Diels-Alder adduct 5 .... S6

2.1.4. 9-(4-Hydroxyphenyl)ethynylanthracene *N*-(2-hydroxyethyl)maleimide 9,10-Diels-Alder adduct 6 .... S7

2.1.5. 9-(4-Hydroxyphenyl)ethynylanthracene *N*-(2-hydroxyethyl)maleimide 9,10-Diels-Alder adduct di(α-bromoisobutyrate) 7 .... S8

2.1.6. 9-(4-Hydroxyphenyl)ethynylanthracene *N*-(2-hydroxyethyl)maleimide 9,10-Diels-Alder adduct dimethacrylate 8 .... S9

2.1.7. 9,10-Bis((trimethylsilyl)ethynyl)anthracene 9 .... S9

2.1.8. 9,10-Bis((trimethylsilyl)ethynyl)anthracene *N*-(2-hydroxyethyl)maleimide 1,4-Diels-Alder adduct 10 .... S10

2.1.9. 9,10-Diethynylanthracene *N*-(2-hydroxyethyl)maleimide 1,4-Diels-Alder adduct 11 S11

2.1.10. 9,10-Di(4-anisylethynyl)anthracene *N*-(2-hydroxyethyl)maleimide 1,4-Diels-Alder adduct 12 .... S11

2.1.11. 9,10-Di(4-anisylethynyl)anthracene *N*-(2-hydroxyethyl)maleimide 1,4-Diels-Alder adduct tri(α-bromoisobutyrate) 13 .... S12

2.2. Reference Compounds 14, 15, and 16 .... S13

2.2.1. 9-((4-Anisyl)ethynyl)anthracene 14 .... S13

2.2.2. 9,10-Bis-((4-anisyl)ethynyl)anthracene *N*-methylmaleimide 1,4-Diels-Alder adduct 15 . ... S14

2.2.3. 9,10-Bis-((4-anisyl)ethynyl)anthracene 16 .... S15

2.2.4. 9,10-Bis-((4-trimethylsilyl)ethynyl)anthracene *N*-methylmaleimide 1,4-Diels-Alder adduct 17 .... S15

2.2.5. 9,10-Bis-4-ethynylanthracene *N*-methylmaleimide 1,4-Diels-Alder adduct 18 .... S16

2.3. Polymerizations of PMA-1, PHMA-1, PHMA-7, and PMA-2 .... S17

2.3.1. SET-LRP towards PMA-1 .... S17

2.3.2. Free radical polymerization towards PHMA-1 .... S18

2.3.3. Free radical polymerization towards reference PHMA-7 .... S18

2.3.4. SET-LRP towards PMA-2 .... S19

3. Determination of Fluorescence Quantum Yields S21

4. Mechanical Scission Experiments S23

4.1. General Procedure for Sonication Experiments S23

4.2. Scission Kinetics of PMA-1 .... S23

4.3. General Procedure for Compression Experiments S24

5. ^{1}H-, ^{13}C-NMR, and HPLC-MS data S25

6. References S33

1. Materials and Methods

1.1. Analytical Instrumentation

NMR spectra were recorded on a 400 MHz (100 MHz for ^{13}C) Varian Mercury VX spectrometer or on a 200 MHz Varian Mercury+ spectrometer at room temperature using residual protonated solvent signals[1] as internal standards (^{1}H: $\delta(CDCl_3)$ = 7.26 ppm, $\delta((CD_3)_2SO)$ = 2.50 ppm, $\delta((CD_3)_2CO)$ = 2.05 ppm; ^{13}C: $\delta(CDCl_3)$ = 77.16 ppm, $\delta((CD_3)_2SO)$ = 39.52 ppm, $\delta((CD_3)_2CO)$ = 29.84 ppm).

Purity and exact mass of the synthesized compounds was determined using a LCQ Fleet (Thermo Finnigan) ion-trap mass spectrometer equipped with a Surveyor autosampler and Surveyor PDA detector (Thermo Finnigan). Solvents were pumped with a flow of 0.2 mLmin^{-1} using a high-pressure gradient system using two LC-10AD pumps (Shimadzu). Before mass analysis, the crude was ran over a reverse phase C18 column (GraceSmart 2 x 50 mm, Grace) using a 2-90% acetonitrile linear gradient in water with 0.1 % formic acid (FA).

Matrix-assisted laser desorption/ionization time-of-flight mass spectrometry (MALDI-TOF MS) was performed on a Autoflex Speed MALDI-MS instrument (Bruker, Bremen, Germany) equipped with a 355 nm Nd:YAG smartbeam laser. MALDI-TOF MS experiments were performed by spotting samples on a MTP 384 target ground steel plate using an α-cyano-4-hydroxycinnamic acid (CHCA) (Fluka, Switzerland) matrix. Samples were 1:1 premixed with CHCA in 50/50 acetonitrile/water supplemented with 0.1% v/v trifluoroacetic acid (TFA). Mass spectra were acquired in reflector positive ion mode by summing spectra from 500 selected laser shots. The MS spectra were calibrated with cesium triiodide of known masses.

Gel permeation chromatography (GPC) was carried out on a Shimadzu Prominence-i LC-2030C 3D system equipped with a RID-20A refractive index detector. TLC was performed on Merck Silica Gel 60 F254 TLC plates with a fluorescent indicator employing a 254 nm UV-lamp for visualization. UV/vis absorption spectroscopy was performed on a PerkinElmer Lambda 900 spectrometer and fluorescence spectroscopy on a PerkinElmer LS 50B fluorescence spectrometer at room temperature.

Sonochemical irradiation experiments were carried out with a Sonics VCX 500 W ultrasonic processor purchased from Sonics & Materials Inc.

1.2. Chemicals and Solvents

Solvents and commercial starting materials were used as supplied. Monomers were purified prior to polymerization by passing over a short column of inhibitor remover. The solvents were dried before use, if necessary, employing an MBraun MB-SPS-800 solvent purification system. Silica gel for chromatography (0.040–0.063 mm, 60 Å) was used for column chromatography. Precursor *N*-(2-hydroxyethyl)maleimide was synthesized according to the literature.[2]

2. Synthetic Procedures and Characterization Data

2.1. Target Compounds 7, 8, and 13

Target compounds **7**, **8**, and **13** were synthesized according to Scheme S1.

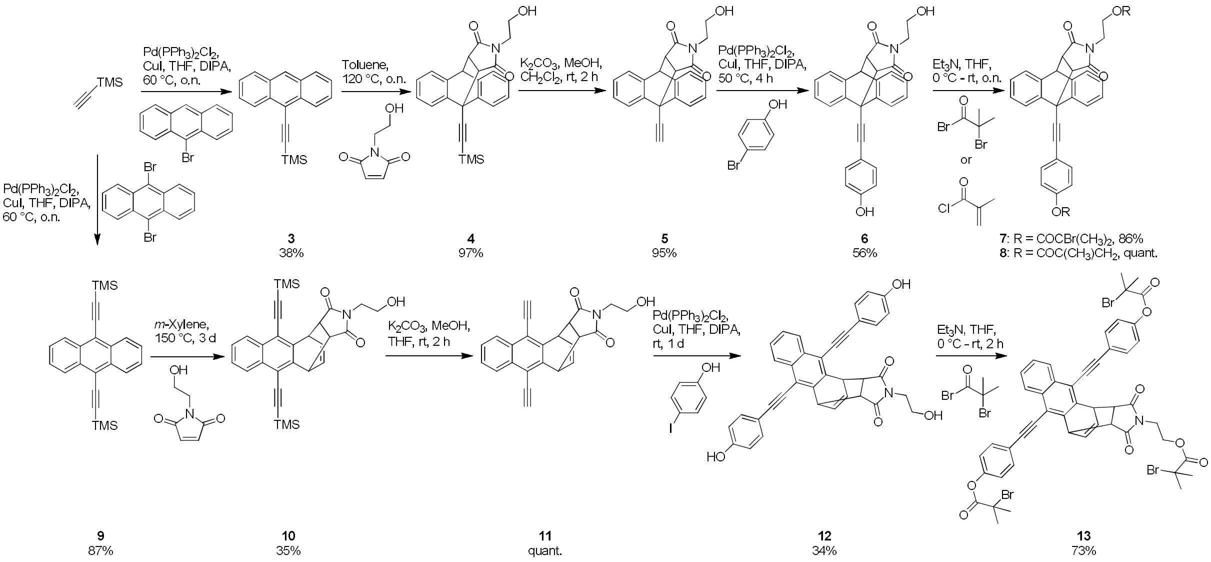

Scheme S1. Synthesis pathway towards target compounds **7**, **8**, and **13**.

2.1.1. (Anthracen-9-ylethynyl)trimethylsilane 3

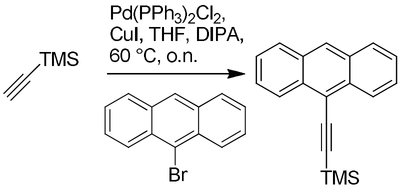

3
38%

9-bromoanthracene (1.500 g, 5.830 mmol), $Pd(PPh_3)_2Cl_2$ (0.205 g, 0.292 mmol), and CuI (0.056 g, 0.292 mmol) were dissolved in dry THF (10 mL) and degassed with Ar. To this solution were added subsequently TMS-acetylene (0.996 mL, 7,000 mmol) and dry diisopropylamine (10 mL) whereupon the solution turned orange and then black. The whole was stirred at 60 °C under Ar o.n. Afterwards, the solution was cooled down and removal of the solvent *in vacuo* and column chromatography (silica, cyclohexane with 1% toluene) yielded a red oil that was recrystallized from MeCN at -30 °C to give (anthracen-9-ylethynyl)trimethylsilane **9** (38% yield) as orange crystals. **^{1}H-NMR (400 MHz, $CDCl_3$):** δ(ppm) = 8.59 (d, ^3J = 8.6 Hz, 2 H, CH_{ar}), 8.43 (s, 1 H, CH_{ar}), 8.01 (d, ^3J = 8.6 Hz, 2 H,

CH_{ar}), 7.61 (m, 2 H, CH_{ar}), 7.52 (m, 2 H, CH_{ar}), 0.45 (s, 9 H, CH_3). **^{13}C-NMR (100 MHz, CDCl$_3$):** δ(ppm) = 132.9, 131.0, 128.6, 127.9, 126.8, 126.7, 125.6, 106.2, 101.5, 95.4, 0.27. **MALDI-TOF-MS:** *m*/*z* = 274.16 (calcd. 274.12 for $C_{19}H_{18}Si^+$).

2.1.2. (Anthracen-9-ylethynyl)trimethylsilane *N*-(2-hydroxyethyl)maleimide 9,10-Diels-Alder adduct 4

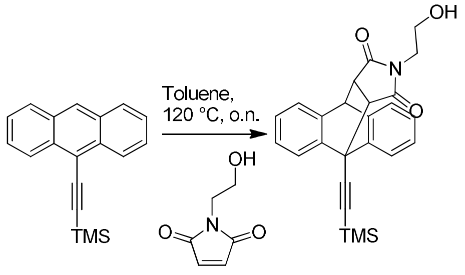

3 **4**
97%

(Anthracen-9-ylethynyl)trimethylsilane **3** (250 mg, 0.911 mmol) and *N*-(2-hydroxyethyl)maleimide (193 mg, 1.366 mmol) were dissolved in toluene (9 mL) and stirred at 120 °C o.n. Evaporation of the solvent *in vacuo* and column chromatography (silica, cyclohexane : EtOAc = 1 : 1) yielded (anthracen-9-ylethynyl)trimethylsilane *N*-(2-hydroxyethyl)maleimide 9,10-Diels-Alder adduct **4** (97% yield) as white foam. **^{1}H-NMR (400 MHz, CDCl$_3$):** δ(ppm) = 7.81 (d, ^3J = 7.4 Hz, 1 H, CH_{ar}), 7.69 (d, ^3J = 7.2 Hz, 1 H, CH_{ar}), 7.40 (d, ^3J = 7.2 Hz, 1 H, CH_{ar}), 7.31 (m, 3 H, CH_{ar}), 7.23 (m, 2 H, CH_{ar}), 4.81 (d, ^3J = 3.1 Hz, 1 H, C*H*), 3.34 (m, 2 H, CH_2), 3.27 (m, 2 H, C*H*), 3.10 (m, 2 H, CH_2), 1.20 (t, ^3J = 6.5 Hz, 1 H, O*H*), 0.42 (s, 9 H, CH_3). **^{13}C-NMR (100 MHz, CDCl$_3$):** δ(ppm) = 176.6, 174.3, 149.4, 147.1, 140.4, 139.9, 138.2, 137.5 127.4, 127.3, 127.2, 126.9, 124.9, 124.1, 123.9, 96.6, 60.3, 50.6, 47.5, 47.3, 45.0, 41.3, 0.16. **HPLC-MS(ESI$^+$):** *m*/*z* = 416.00 (calcd. 416.16 for $C_{25}H_{26}NO_3Si^+$). **MALDI-TOF-MS:** *m*/*z* = 438.16 (calcd. 438.15 for $C_{25}H_{25}NO_3SiNa^+$).

2.1.3. 9-Ethynylanthracene *N*-(2-hydroxyethyl)maleimide 9,10-Diels-Alder adduct 5

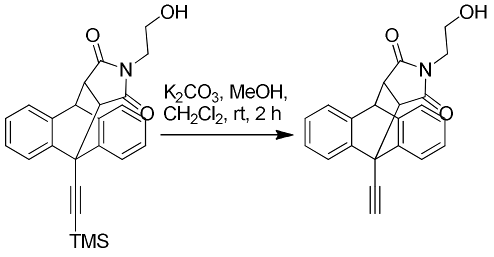

4 **5**
95%

To a degassed solution of K_2CO_3 (80 mg, 0.578 mmol) in MeOH (2 mL) was added a degassed solution of (anthracen-9-ylethynyl)trimethylsilane *N*-(2-hydroxyethyl)maleimide 9,10-Diels-Alder adduct **4** (300 mg, 0,722 mmol) in a mixture of CH_2Cl_2 (2 mL) and MeOH (2 mL) dropwise at rt under Ar and was stirred at that temperature for 2 h. Subsequently, the solution was diluted with water and extracted with Et_2O. The combined organic layers were dried over anhyd. $MgSO_4$ and removal of the volatiles *in vacuo* yielded pure 9-ethynylanthracene *N*-(2-hydroxyethyl)maleimide 9,10-Diels-Alder adduct **5** (95% yield) as white foam. **^{1}H-NMR (400 MHz, $CDCl_3$):** δ(ppm) = 7.85 (d, ^3J = 7.4 Hz, 1 H, CH_{ar}), 7.74 (d, ^3J = 6.8 Hz, 1 H, CH_{ar}), 7.41 (d, ^3J = 6.8 Hz, 1 H, CH_{ar}), 7.31 (m, 3 H, CH_{ar}), 7.25 (m, 2 H, CH_{ar}), 4.82 (d, ^3J = 3.1 Hz, 1 H, C*H*), 3.32 (m, 4 H, C*H*, CH_2), 3.18 (s, 1 H, C*H*), 3.11 (m, 2 H, CH_2), 1.16 (t, ^3J = 6.5 Hz, 1 H, O*H*). **^{13}C-NMR (100 MHz, $CDCl_3$):** δ(ppm) = 176.4, 174.7, 140.1, 139.8, 137.8, 137.4 127.6, 127.5, 127.2, 126.9, 125.0, 123.94, 123.92, 123.7, 79.5, 78.6, 60.0, 50.5, 47.4, 46.5, 44.9, 41.3. **HPLC-MS(ESI$^+$):** m/z = 344.00 (calcd. 344.12 for $C_{22}H_{18}NO_3^+$).

2.1.4. 9-(4-Hydroxyphenyl)ethynylanthracene *N*-(2-hydroxyethyl)maleimide 9,10-Diels-Alder adduct 6

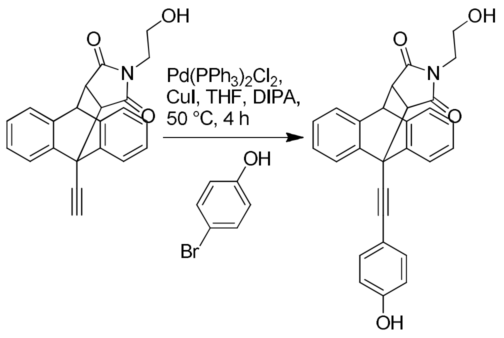

6
56%

9-Ethynylanthracene *N*-(2-hydroxyethyl)maleimide 9,10-Diels-Alder adduct **5** (180 mg, 0.524 mmol), 4-bromophenol (150 mg, 0.867 mmol), $Pd(PPh_3)_2Cl_2$ (18 mg, 0.026 mmol), and CuI (5 mg, 0.026 mmol) were dissolved in dry THF (1.5 mL) and degassed. To this solution was added dry diisopropylamine (1.5 mL) whereupon the solution turned black. The whole was stirred at 50 °C under Ar for 4 h. Afterwards, the solution was cooled down, diluted with THF, and filtered to remove the HBr salt. Subsequent removal of the solvent *in vacuo* and column chromatography (silica, cyclohexane : EtOAc = 1 : 1) yielded 9-(4-hydroxyphenyl)ethynylanthracene *N*-(2-hydroxyethyl)maleimide 9,10-Diels-Alder adduct **6** (56% yield) as off-white solid. **^{1}H-NMR**

(400 MHz, $(CD_3)_2SO$): δ(ppm) = 9.93 (s, 1 H, O*H*), 7.76 (d, ^3J = 7.0 Hz, 1 H, CH_{ar}), 7.55 (m, 4 H, CH_{ar}), 7.27 (m, 5 H, CH_{ar}), 6.87 (d, ^3J = 8.6 Hz, 2 H, CH_{ar}), 4.84 (m, 1 H, C*H*), 4.62 (m, 1 H, O*H*) 3.33 (m, 2 H, C*H*), 3.01 (t, ^3J = 7.4 Hz, 2 H, CH_2), 2.56 (m, 2 H, CH_2). **HPLC-MS(ESI$^+$):** *m*/*z* = 436.17 (calcd. 436.15 for $C_{28}H_{22}NO_4^+$).

2.1.5. 9-(4-Hydroxyphenyl)ethynylanthracene *N*-(2-hydroxyethyl)maleimide 9,10-Diels-Alder adduct di(α-bromoisobutyrate) 7

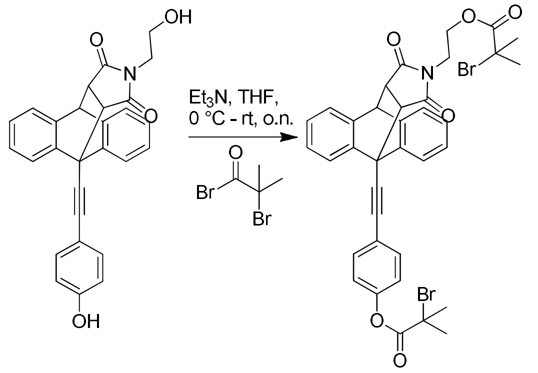

6 **7**
86%

9-(4-Hydroxyphenyl)ethynylanthracene *N*-(2-hydroxyethyl)maleimide 9,10-Diels-Alder adduct **6** (110 mg, 0.253 mmol) and Et_3N (0.072 mL, 0.518 mmol) were placed in a Schlenk flask under Ar. To this was added dry THF (2 mL) and the solution then cooled with an ice bath. Subsequently, α-bromoisobutyryl bromide (0.064 mL, 0.518 mmol) was added dropwise and the final solution was left to stir o.n. at rt. All volatiles were removed *in vacuo* and subsequent column chromatography (silica, cyclohexane : EtOAc = 1 : 1 with 1% toluene) yielded 9-(4-hydroxyphenyl)ethynylanthracene *N*-(2-hydroxyethyl)maleimide 9,10-Diels-Alder adduct di(α-bromoisobutyrate) **7** (86% yield) as yellowish foam. **^{1}H-NMR (400 MHz, $CDCl_3$):** δ(ppm) =7.87 (d, ^3J = 8.6 Hz, 1 H, CH_{ar}), 7.82 (d, ^3J = 8.6 Hz, 2 H, CH_{ar}), 7.3 (d, ^3J = 8.60 Hz, 1 H, CH_{ar}), 7.43 (d, ^3J = 7.0 Hz, 1 H, CH_{ar}), 7.31 (m, 2 H, CH_{ar}), 7.24 (m, 6 H, CH_{ar}), 4.83 (m, 1 H, C*H*), 3.68 (m, 1 H, CH_2), 3.60 (m, 1 H, CH_2), 3.41 (m, 2 H, CH_2), 3.33 (d, ^3J = 1.6 Hz, 2 H, C*H*), 2.11 (s, 6 H, CH_3), 1.90 (s, 6 H, CH_3). **^{13}C-NMR (100 MHz, $CDCl_3$):** δ(ppm) = 175.7, 173.8, 171.2, 169.9, 150.8, 140.7, 140.0, 137.8, 137.1, 133.3, 127.6, 127.3, 127.2, 126.9, 124.9, 123.98, 123.96, 123.7, 121.3, 120.8, 90.4, 84.4, 62.1, 55.5, 55.2, 50.8, 47.6, 47.1, 44.9, 36.7, 30.6, 30.5. **HPLC-MS(ESI$^+$):** *m*/*z* = 732.17 (calcd. 732.06 for $C_{36}H_{32}Br_2NO_6^+$). 32

2.1.6. 9-(4-Hydroxyphenyl)ethynylanthracene *N*-(2-hydroxyethyl)maleimide 9,10-Diels-Alder adduct dimethacrylate 8

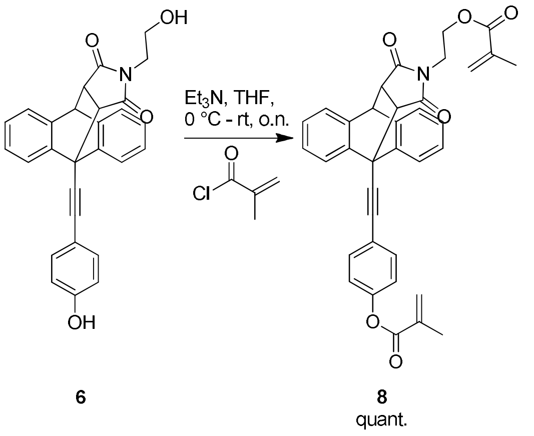

9-(4-Hydroxyphenyl)ethynylanthracene *N*-(2-hydroxyethyl)maleimide 9,10-Diels-Alder adduct **6** (20 mg, 0.046 mmol) and Et_3N (13 µL, 0.096 mmol) were dissolved in dry THF (0.5 mL) under Ar and the solution was then cooled with an ice bath. Subsequently, methacryloyl chloride (9.3 µL, 0.096 mmol) was added and the final solution was left to stir o.n. at rt. Subsequently, the reaction mixture was passed through a plug of silica and all volatiles were removed *in vacuo* to yield pure 9-(4-hydroxyphenyl)ethynylanthracene *N*-(2-hydroxyethyl)maleimide 9,10-Diels-Alder adduct dimethacrylate **8** (quant. yield) as colorless wax that was stored at -30 °C in a Schlenk flask under Ar and could not be characterized further due to its instable nature. **HPLC-MS(ESI$^+$):** *m*/*z* = 572.17 (calcd. 572.20 for $C_{36}H_{30}NO_6^+$).

2.1.7. 9,10-Bis((trimethylsilyl)ethynyl)anthracene 9

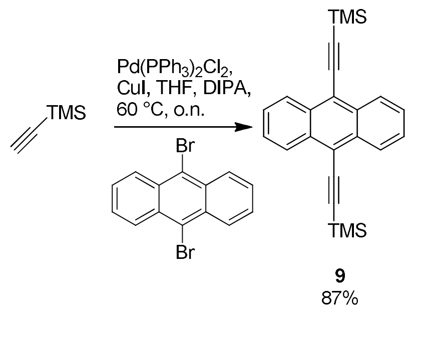

9,10-Dibromoanthracene (1 g, 2.980 mmol), $Pd(PPh_3)_2Cl_2$ (0.209 g, 0.298 mmol), and CuI (0.057 g, 0.298 mmol) were dissolved in dry THF (8 mL) and degassed. To this solution were added subsequently TMS-acetylene (0.932 mL, 6.550 mmol) and dry diisopropylamine (8 mL) whereupon the solution turned black. The whole was stirred at 60 °C under Ar o.n. Afterwards, the solution was

cooled down, diluted with THF, and filtered through a plug of silica to remove the HBr salt. Subsequent removal of the solvent *in vacuo* and column chromatography (silica, cyclohexane with 1% toluene) yielded 9,10-bis((trimethylsilyl)ethynyl)anthracene **9** (87% yield) as orange solid. **^{1}H-NMR (400 MHz, $CDCl_3$):** δ(ppm) = 8.60 (d×d, ^{3}J = 6.8, 3.7 Hz, 4 H, CH_{ar}), 7.63 (d×d, ^{3}J = 6.8, 3.3 Hz, 4 H, CH_{ar}), 0.45 (s, 18 H, CH_3). **^{13}C-NMR (100 MHz, $CDCl_3$):** δ(ppm) = 132.2, 127.2, 126.8, 118.4, 108.2, 101.5, 0.2. **HPLC-MS(ESI$^+$):** m/z = 370.33 (calcd. 370.16 for $C_{24}H_{26}Si_2^+$).

2.1.8. 9,10-Bis((trimethylsilyl)ethynyl)anthracene *N*-(2-hydroxyethyl)maleimide 1,4-Diels-Alder adduct 10

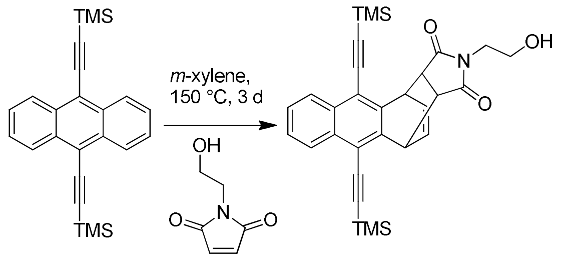

9,10-Bis((trimethylsilyl)ethynyl)anthracene **9** (250 mg, 0.675 mmol) and *N*-(2-hydroxyethyl)maleimide (238 mg, 1.686 mmol) were dissolved in *m*-xylene (5 mL) and stirred at 150 °C for 3 d. Afterwards, the reaction was cooled to rt, diluted with THF, and subsequently all solvents were removed *in vacuo*. Column chromatography (silica, heptane : EtOAc = 2 : 1 with 1% toluene) yielded 9,10-bis((trimethylsilyl)ethynyl)anthracene *N*-(2-hydroxyethyl)maleimide 1,4-Diels-Alder adduct **10** (35% yield) as yellowish solid. **^{1}H-NMR (400 MHz, $CDCl_3$):** δ(ppm) = 8.30 (d×d, ^{3}J = 6.1, 2.9 Hz, 2 H, CH_{ar}), 7.60 (d×d, ^{3}J = 5.7, 2.9 Hz, 2 H, CH_{ar}), 6.60 (m, 2 H, C*H*), 5.12 (m, 2 H, C*H*), 3.75 (m, 4 H, CH_2), 3.09 (s, 2 H, C*H*), 2.25 (br. s., 1 H, O*H*), 0.41 (s, 18 H, CH_3). **^{13}C-NMR (100 MHz, $CDCl_3$):** δ(ppm) = 177.6, 141.7, 133.7, 131.5, 127.2, 126.6, 115.9, 105.7, 99.8, 60.7, 45.2, 41.7, 40.2, 0.1. **HPLC-MS(ESI$^+$):** m/z = 511.92 (calcd. 512.20 for $C_{30}H_{34}NO_3Si_2^+$).

2.1.9. 9,10-Diethynylanthracene *N*-(2-hydroxyethyl)maleimide 1,4-Diels-Alder adduct 11

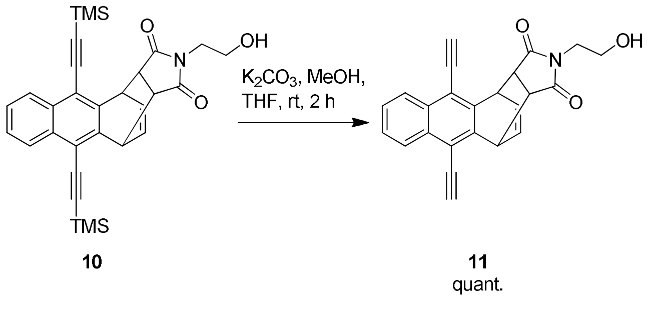

To a solution of K_2CO_3 (52 mg, 0.375 mmol) in MeOH (1 mL) was added a solution of 9,10-bis((trimethylsilyl)ethynyl)anthracene *N*-(2-hydroxyethyl)maleimide 1,4-Diels-Alder adduct **10** (120 mg, 0.234 mmol) in a mixture of MeOH (1 mL) and THF (2 mL). The whole was degassed by bubbling through Ar and stirred at rt for 2 h. Subsequently, the solution was diluted with water and the aqueous phase extracted with EtOAc. The combined organic layers were dried over anhyd. $MgSO_4$ and the solution concentrated *in vacuo*. The product is temperature as well as light sensitive and hence the final amounts of solvent were removed *in vacuo* in a dry Schlenk tube under Ar. Subsequently, 9,10-diethynylanthracene *N*-(2-hydroxyethyl)maleimide 1,4-Diels-Alder adduct **11** (quant. yield) was received as white powder that was used immediately and without further purification. **HPLC-MS(ESI$^+$):** m/z = 367.92 (calcd. 368.12 for $C_{24}H_{18}NO_3^+$).

2.1.10. 9,10-Di(4-anisylethynyl)anthracene *N*-(2-hydroxyethyl)maleimide 1,4-Diels-Alder adduct 12

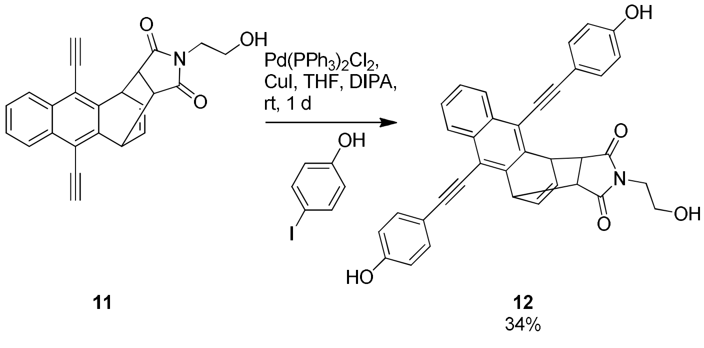

9,10-Diethynylanthracene *N*-(2-hydroxyethyl)maleimide 1,4-Diels-Alder adduct **11** (86 mg, 0.234 mmol), 4-iodophenol (113 mg, 0.515 mmol), $Pd(PPh_3)_2Cl_2$ (17 mg, 0.023 mmol), and CuI (5 mg, 0.023 mmol) were dissolved in dry THF (1 mL) and degassed. To this solution was added dry diisopropylamine (1 mL) whereupon the solution turned black. The whole was stirred at rt under Ar

o.n. Afterwards, the solution was diluted with THF and removal of the solvent *in vacuo* and column chromatography (silica, heptane : EtOAc = 1 : 4) yielded 9,10-di(4-anisylethynyl)anthracene *N*-(2-hydroxyethyl)maleimide 1,4-Diels-Alder adduct **12** (34% yield) as a yellowish solid. **^{1}H-NMR (400 MHz, ((CD$_3$)$_2$CO):** δ(ppm) = 8.47 (m, 2 H, CH_{ar}), 7.67 (m, 6 H, CH_{ar}), 6.99 (m, 4 H, CH_{ar}), 6.66 (m, 2 H, C*H*), 5.15 (m, 2 H, C*H*), 3.59 (m, 4 H, CH_2), 3.23 (s, 2 H, C*H*). Hydroxyl protons are not visible. ^{13}C-NMR could not be recorded due to low solubility in the common deuterated solvents. **HPLC-MS(ESI$^+$):** m/z = 551.83 (calcd. 552.17 for $C_{36}H_{26}NO_5^+$).

2.1.11. 9,10-Di(4-anisylethynyl)anthracene *N*-(2-hydroxyethyl)maleimide 1,4-Diels-Alder adduct tri(α-bromoisobutyrate) 13

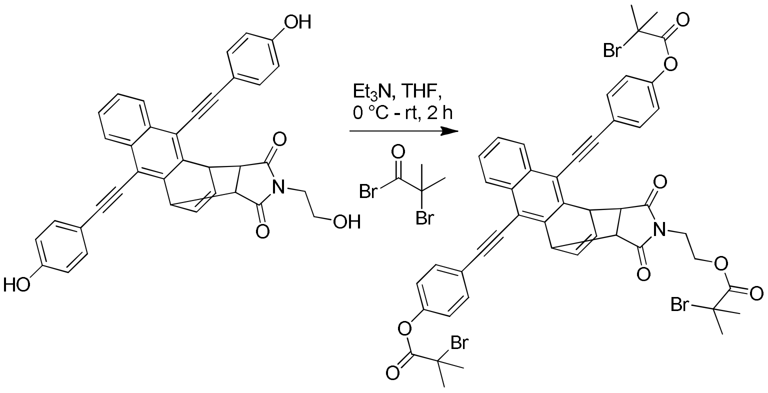

9,10-Di(4-anisylethynyl)anthracene *N*-(2-hydroxyethyl)maleimide 1,4-Diels-Alder adduct **12** (44 mg, 0.080 mmol) and Et$_3$N (0.11 mL, 0.798 mmol) were dissolved in dry THF (3 mL) in a Schlenk flask under Ar. Subsequently, α-bromoisobutyryl bromide (0.10 mL, 0.798 mmol) was added dropwise and the final solution was left to stir for 2 h at rt. Afterwards, the mixture was diluted with THF and filtered through a pad of silica to remove the HBr salt. All volatiles were removed *in vacuo* and the resulting solid was slurried in MeOH, filtered, and washed multiple times with water and MeOH to remove residual reactants and to eventually yield pure 9,10-di(4-anisylethynyl)anthracene *N*-(2-hydroxyethyl)maleimide 1,4-Diels-Alder adduct tri(α-bromoisobutyrate) **13** (73% yield) as yellowish solid. **^{1}H-NMR (400 MHz, CDCl$_3$):** δ(ppm) = 8.43 (d×d, ^3J = 6.4, 3.2 Hz, 2 H, CH_{ar}), 7.79 (d, ^3J = 8.6 Hz, 4 H, CH_{ar}), 7.65 (d×d, ^3J = 6.4, 3.2 Hz, 2 H, CH_{ar}), 7.25 (d, ^3J = 8.6 Hz, 4 H, CH_{ar}), 6.67 (m, 2 H, C*H*), 5.21 (m, 2 H, CH_{ar}), 4.35 (t, ^3J = 5.0 Hz, 2 H, CH_2), 3.84 (t, ^3J = 5.0 Hz, 2 H, CH_2), 3.16 (s, 2 H, C*H*), 2.12 (s, 12 H, CH_3), 1.94 (s, 6 H, CH_3). **^{13}C-NMR (100 MHz, CDCl$_3$):** δ(ppm) = 177.7,

176.6, 171.2, 140.7, 133.9, 133.1, 131.9, 130.2, 127.5, 123.1, 121.5, 116.0, 113.4, 105.6, 98.7, 75.6, 70.2, 70.1, 50.36, 50.33, 40.2, 30.66, 30.62. **HPLC-MS(ESI$^+$):** *m*/*z* = 998.92 (calcd. 999.03 for $C_{48}H_{40}Br_3NO_8^+$).

2.2. Reference Compounds 14, 15, and 16

Reference compounds **14**, **15**, and **16** were synthesized according to Scheme S2.

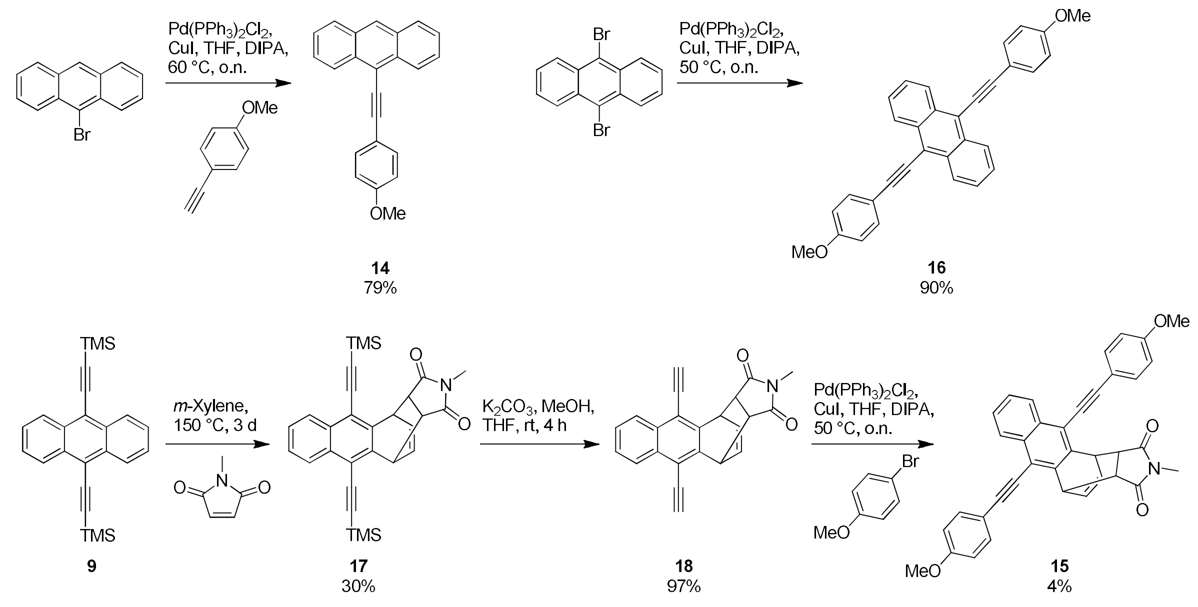

Scheme S2. Synthesis pathway towards reference compounds **14**, **15**, and **16**.

2.2.1. 9-((4-Anisyl)ethynyl)anthracene 14

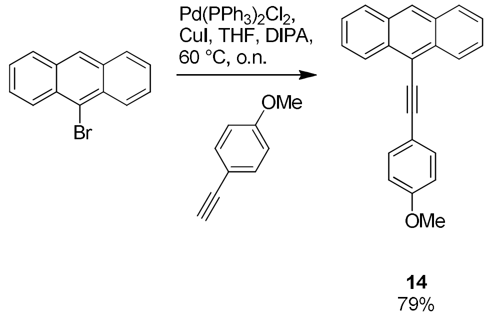

14
79%

9-Bromoanthracene (500 mg, 1.945 mmol), 4-ethynylanisole (257 mg, 1.945 mmol), $Pd(PPh_3)_2Cl_2$ (68 mg, 0.097 mmol), and CuI (19 mg, 0.097 mmol) were dissolved in dry THF (5 mL) and degassed. To this solution was added dry diisopropylamine (5 mL) whereupon the solution turned black. The whole was stirred at 60 °C under Ar o.n. Removal of the solvent *in vacuo* and column chromatography (silica, pentane : EtOAc = 9 : 1 with 1% toluene) yielded 9-((4-anisyl)ethynyl)anthracene **14** (79%

yield) as fluffy, yellow solid. **^{1}H-NMR (400 MHz, $CDCl_3$):** δ(ppm) = 8.68 (d, ^3J = 9.0 Hz, 2 H, CH_{ar}), 8.42 (s, 1 H, CH_{ar}), 8.03 (d, ^3J = 8.6 Hz, 2 H, CH_{ar}), 7.73 (d, ^3J = 9.0 Hz, 2 H, CH_{ar}), 7.60 (m, 2 H, CH_{ar}), 7.51 (m, 2 H, CH_{ar}), 6.99 (d, ^3J = 8.6 Hz, 2 H), 3.89 (s, 3 H, CH_3). **^{13}C-NMR (100 MHz, $CDCl_3$):** δ(ppm) = 159.8, 133.1, 132.4, 131.2, 128.6, 127.2, 126.8, 126.4, 125.6, 123.3, 123.2, 119.1, 117.7, 114.2, 55.4. **HPLC-MS(ESI$^+$):** m/z = 309.25 (calcd. 309.12 for $C_{23}H_{17}O^+$).

2.2.2. 9,10-Bis-((4-anisyl)ethynyl)anthracene *N*-methylmaleimide 1,4-Diels-Alder adduct 15

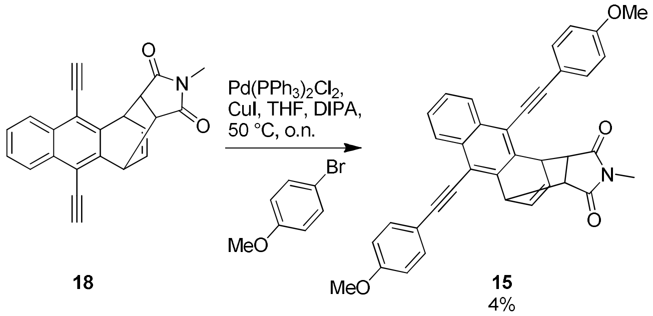

9,10-Diethynylanthracene *N*-methylmaleimide 1,4-Diels-Alder adduct **18** (70 mg, 0.207 mmol), 4-bromoanisole (54 µL, 0.433 mmol), $Pd(PPh_3)_2Cl_2$ (15 mg, 0.021 mmol), and CuI (4 mg, 0.021 mmol) were dissolved in dry THF (0.5 mL) and degassed. To this solution was added dry diisopropylamine (0.5 mL) whereupon the solution turned black. The whole was stirred at 50 °C under Ar o.n. Afterwards, the solution was cooled down, diluted with THF, and filtered to remove the HBr salt. Subsequent removal of the solvent *in vacuo* and column chromatography (silica, cyclohexane : EtOAc = 1 : 1) yielded 9,10-bis-((4-anisyl)ethynyl)anthracene *N*-methylmaleimide 1,4-Diels-Alder adduct **15** (4% yield) as a yellowish solid. **^{1}H-NMR (400 MHz, $CDCl_3$):** δ(ppm) = 8.43 (d×d, ^3J = 6.3, 3.5 Hz, 2 H, CH_{ar}), 7.69 (d, ^3J = 8.2 Hz, 4 H, CH_{ar}), 7.62 (d×d, ^3J = 6.3, 3.5 Hz, 2 H, CH_{ar}), 6.99 (d, ^3J = 8.2 Hz, 4 H, CH_{ar}), 6.62 (m, 2 H, CH), 5.22 (m, 2 H, CH), 3.90 (s, 6 H, CH_3), 3.13 (s, 2 H, CH), 3.00 (s, 3 H, CH_3). **^{13}C-NMR (100 MHz, $CDCl_3$):** δ(ppm) = 177.2, 160.1, 144.5, 133.9, 133.4, 131.6, 127.0, 126.8, 125.8, 114.2, 108.5, 99.6, 66.4, 55.4, 45.5, 30.9, 24.9. **HPLC-MS(ESI$^+$):** m/z = 550.75 (calcd. 550.19 for $C_{37}H_{28}NO_4^+$).

2.2.3. 9,10-Bis-((4-anisyl)ethynyl)anthracene 16

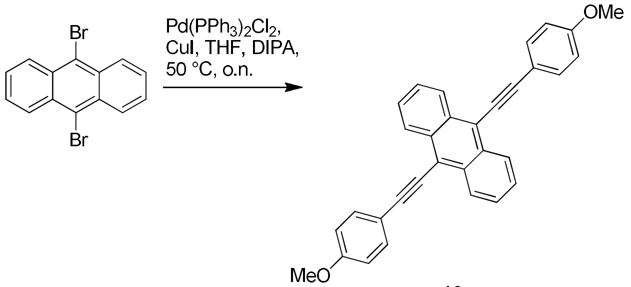

16
90%

9,10-Dibromoanthracene (580 mg, 1.726 mmol), $Pd(PPh_3)_2Cl_2$ (121 mg, 0.173 mmol), and CuI (33 mg, 0.173 mmol) were dissolved in dry THF (6 mL) and degassed. To this solution were added subsequently 4-ethynylanisole (502 mg, 3.80 mmol) and dry diisopropylamine (6 mL) whereupon the solution turned black. The whole was stirred at 50 °C under Ar o.n. Afterwards, the solution was cooled down, diluted with THF, and filtered through a plug of silica to remove the HBr salt. Subsequently the solvent was removed *in vacuo* and the residue re-dispersed in MeCN. Filtration and washing of the residue with MeCN (6×) yielded 9,10-bis((4-anisyl)ethynyl)anthracene **16** (90% yield) as orange solid. **^{1}H-NMR (400 MHz, $CDCl_3$):** δ(ppm) = 8.69 (m, 4 H, CH_{ar}), 7.73 (d, ^3J = 8.6 Hz, 2 H, CH_{ar}), 7.63 (m, 4 H, CH_{ar}), 7.00 (d, ^3J = 8.6 Hz, 4 H, CH_{ar}), 3.90 (s, 6 H, CH_3). **^{13}C-NMR (100 MHz, $CDCl_3$):** δ(ppm) = 162.7, 133.2, 132.0, 128.7, 127.3, 126.6, 125.9, 118.2, 115.3, 114.2, 55.4. **HPLC-MS(ESI$^+$):** m/z = 438.33 (calcd. 438.16 for $C_{32}H_{22}O_2^+$).

2.2.4. 9,10-Bis-((4-trimethylsilyl)ethynyl)anthracene *N*-methylmaleimide 1,4-Diels-Alder adduct 17

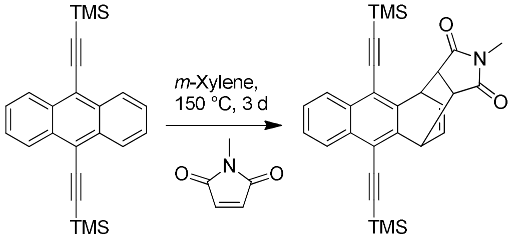

9 **17**
30%

9,10-Bis((trimethylsilyl)ethynyl)anthracene (500 mg, 1.349 mmol) and *N*-methylmaleimide (225 mg, 2.024 mmol) were dissolved in *m*-xylene (10 mL) and stirred at 150 °C for 3 d. After that time, the reaction was cooled to rt and precipitated in H_2O/MeOH. Filtration yielded the crude product that was

purified by column chromatography (silica, cyclohexane : EtOAc = 9 : 1 with 1% toluene) and subsequently gave 9,10-bis-((4-trimethylsilyl)ethynyl)anthracene *N*-methylmaleimide 1,4-Diels-Alder adduct **17** (30% yield) as white powder. **^{1}H-NMR (400 MHz, $CDCl_3$):** δ(ppm) = 8.31 (m, 2 H, CH_{ar}), 7.60 (m, 2 H, CH_{ar}), 6.56 (m, 2 H, C*H*), 5.11 (m, 2 H, C*H*), 3.05 (s, 2 H, C*H*), 2.98 (s, 3 H, CH_3), 0.4 (s, 18 H, CH_3). **^{13}C-NMR (100 MHz, $CDCl_3$):** δ(ppm) = 177.0, 141.8, 133.7, 131.5, 127.2, 126.6, 115.9, 99.8, 64.8, 45.3, 40.1, 24.9, 0.1. **HPLC-MS(ESI$^+$):** m/z = 481.92 (calcd. 482.19 for $C_{29}H_{32}NO_2Si_2^+$).

2.2.5. 9,10-Bis-4-ethynylanthracene *N*-methylmaleimide 1,4-Diels-Alder adduct 18

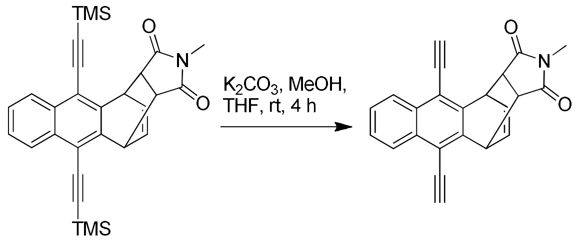

17 **18** 97%

To a solution of K_2CO_3 (46 mg, 0.332 mmol) in MeOH (1 mL) was added a solution of 9,10-bis-((4-trimethylsilyl)ethynyl)anthracene *N*-methylmaleimide 1,4-Diels-Alder adduct **17** (100 mg, 0.208 mmol) in a mixture of MeOH (1 mL) and THF (4 mL). The whole was stirred at rt for 4 h. Subsequently, the solution was diluted with water and MeOH, the precipitate filtered and washed with water and MeOH, and finally dried to yield pure 9,10-bis-4-ethynylanthracene *N*-methylmaleimide 1,4-Diels-Alder adduct **18** (97% yield) as white powder. The product is temperature as well as light sensitive and hence was used without further purification. **HPLC-MS(ESI$^+$):** m/z = 337.92 (calcd. 338.11 for $C_{23}H_{16}NO_2^+$).

2.3. Polymerizations of PMA-1, PHMA-1, PHMA-7, and PMA-2

2.3.1. SET-LRP towards PMA-1

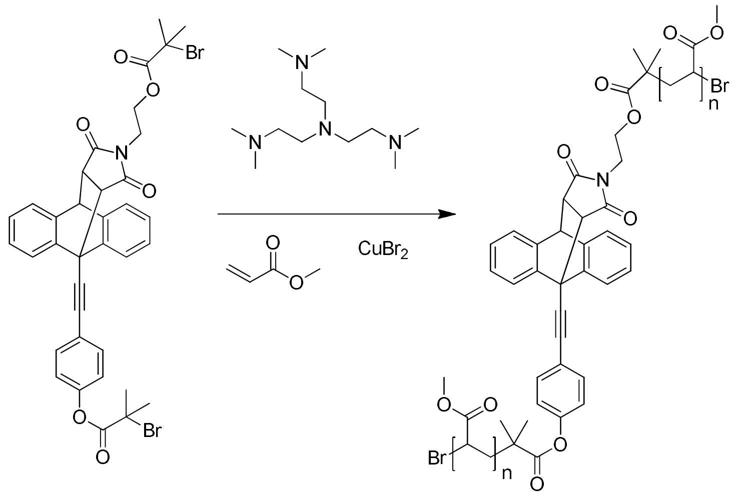

9-(4-Hydroxyphenyl)ethynylanthracene *N*-(2-hydroxyethyl)maleimide 9,10-Diels-Alder adduct di(α-bromoisobutyrate) **7** (37 mg, 0.05 mmol), Me_6TREN (2 µl, 8,00 µmol), methyl acrylate (3.2 mL, 35.00 mmol), and $CuBr_2$ (0.56 mg, 2,50 µmol) were dissolved in DMSO (3.2 mL) in a Schlenk flask and degassed by 3 consecutive freeze-pump-thaw cycles. During that time, copper wire (0.5 cm) was activated in conc. HCl, subsequently washed with water and acetone, and dried. This was then added to the solution and the polymerization was allowed to run at rt for 6 h. Then, the viscous solution was diluted with THF, passed through a plug of basic Al_2O_3, and after concentration *in vacuo*, added dropwise to stirred, ice-cold MeOH. MeOH was decanted and the viscous polymer redissolved in THF whereupon after concentration *in vacuo* it was again precipitated dropwise in fresh MeOH. After repeating the precipitation process 3 times, the polymer was dried in vacuo and received as a ductile white solid (48%). **M_n (GPC):** 60 kD, **M_n (^{1}H-NMR):** 65 kD. **M_w/M_n (GPC):** 1.12. (compare Figure S1)

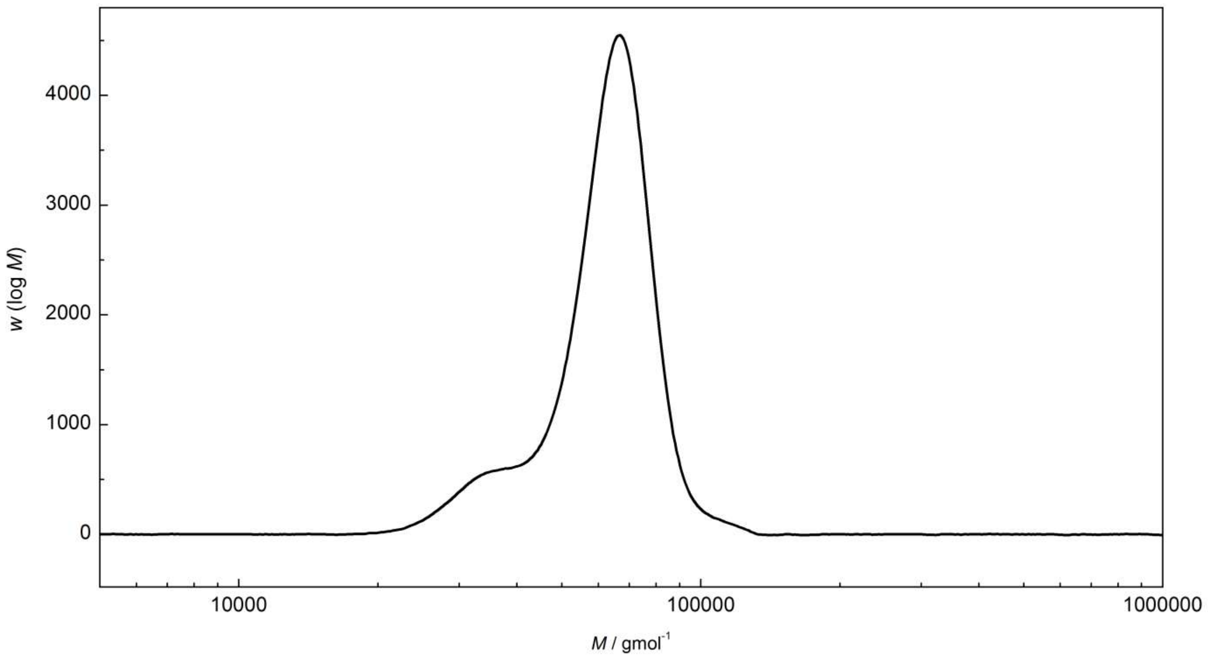

Figure S1. Differential molecular weight distribution of GPC RI chromatogram of **PMA-1**.

2.3.2. Free radical polymerization towards PHMA-1

Hexyl methacrylate (2.0 mL, 10.00 mmol) was mixed with dibenzoyl peroxide (21 mg, 0.066 mmol), ethylene glycol dimethacrylate (18 µL, 0.098 mmol), and 9-(4-hydroxyphenyl)ethynylanthracene *N*-(2-hydroxyethyl)maleimide 9,10-Diels-Alder adduct dimethacrylate **8** (1.1 mg, 0.002 mmol) in a vial and flushed with Ar for 30 s. The mixture was pipetted into a PTFE mold and put in an oven at 60 °C for 6 h under N_2.

2.3.3. Free radical polymerization towards reference PHMA-7

Hexyl methacrylate (2.0 mL, 10.00 mmol) was mixed with dibenzoyl peroxide (21 mg, 0.066 mmol), ethylene glycol dimethacrylate (19 µL, 0.1 mmol), and 9-(4-hydroxyphenyl)ethynylanthracene *N*-(2-hydroxyethyl)maleimide 9,10-Diels-Alder adduct di(α-bromoisobutyrate) **7** (1.5 mg, 0.002 mmol) in a vial and flushed with Ar for 30 s. The mixture was pipetted into a PTFE mold and put in an oven at 60 °C for 6 h under N_2.

2.3.4. SET-LRP towards PMA-2

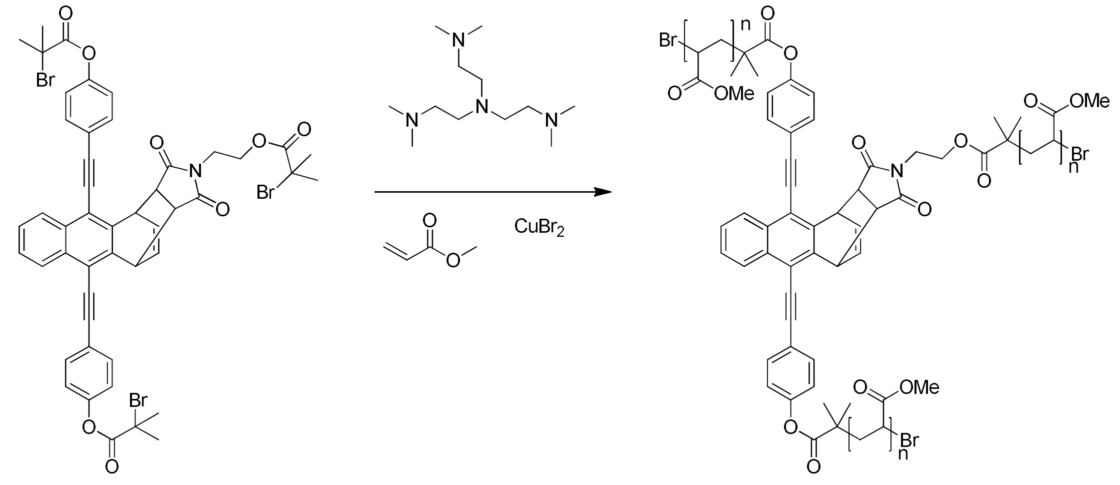

13

PMA-2
69%

9,10-Di(4-anisylethynyl)anthracene *N*-(2-hydroxyethyl)maleimide 1,4-Diels-Alder adduct tri(α-bromoisobutyrate) **13** (19 mg, 0.019 mmol), Me_6TREN (0.8 µL, 3.04 µmol), methyl acrylate (1.9 mL, 20.93 mmol), and $CuBr_2$ (0.2 mg, 0.95 µmol) were dissolved in DMF (4 mL) in a Schlenk tube and degassed by 3 consecutive freeze-pump-thaw cycles. During that time, copper wire (0.5 cm) was activated in conc. H_2SO_4, subsequently washed with water and acetone, and dried. The wire was then added to the solution and the polymerization was allowed to run at rt for 6 h. The solution was then diluted with THF, passed through a plug of basic Al_2O_3, and precipitated in ice-cold MeOH. MeOH was decanted and the formed precipitate repeatedly washed with ice-cold MeOH. Afterwards, the polymer was dried *in vacuo* and received as a ductile, yellowish solid (69%). **M_n (GPC):** 112 kD, **M_n (^{1}H-NMR):** 130 kD. **M_w/M_n (GPC):** 1.17. (compare Figure S2)

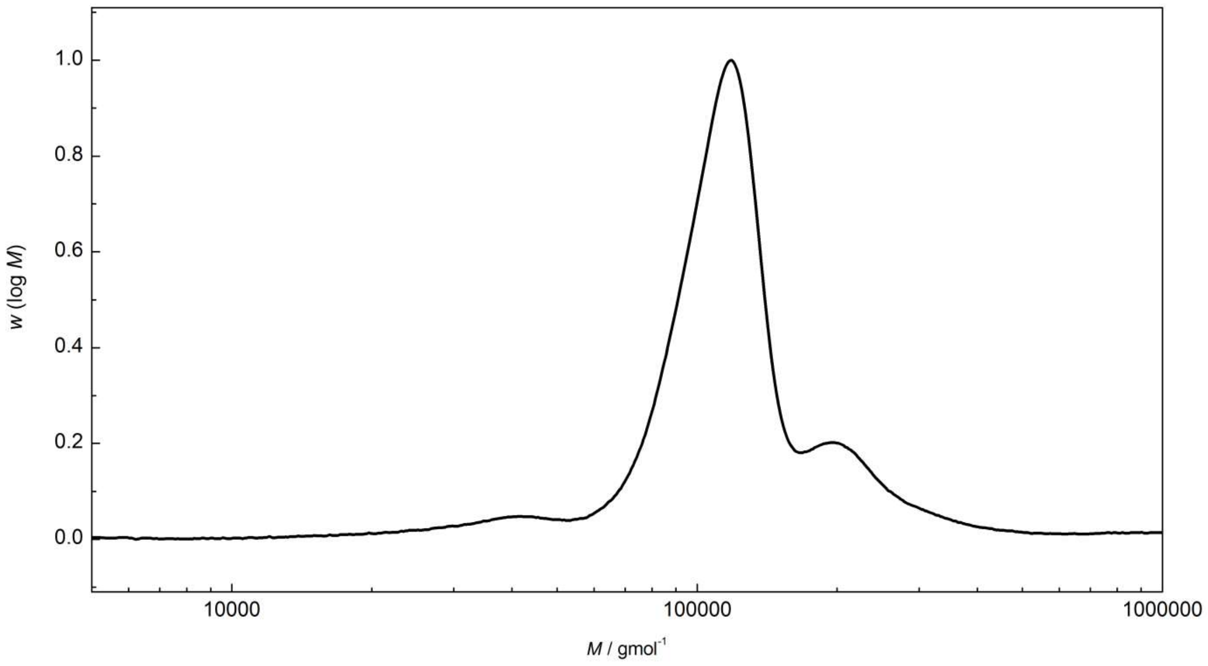

Figure S2. Differential molecular weight distribution of GPC RI chromatogram of **PMA-2**.

3. Determination of Fluorescence Quantum Yields

Fluorescence quantum yields ϕ_x were determined by recording the emission fluorescence spectra of degassed analyte (and standard) solutions with different known absorbances of $A < 0.1$ at and above the excitation wavelength.[3,4] Plotting of absorbance A at the emission wavelength vs. the integrated fluorescence intensity I_{int} and subsequent linear regression results in a slope m allowing the calculation of ϕ_x according to

$$\phi_x = \phi_{ref} \frac{m_x \eta_x^2}{m_{ref} \eta_{ref}^2}$$

where ϕ_{ref} is the fluorescence quantum yield of a reference compound well described in the literature and η is the refractive index of the employed solvent.

Fluorescence quantum yields were determined relative to 9,10-diphenylanthracene (DPA) in cyclohexane assuming its fluorescence quantum yield to ϕ_f to 0.97.[5] The excitation wavelength used was λ_{exc} = 372 nm, excitation and emission slit widths were 4 nm on fluorescence as well as on absorption spectrometer. The solutions were degassed by bubbling though Ar for roughly 30 s prior to measurement. Absorption and emission spectra of DPA are depicted in Figure S3. The absorption and emission spectra for reference **14** are combined in Figure S4. Plots of A vs. I_{int} including linear regressions of DPA as well as **14** can be found in Figure S5.

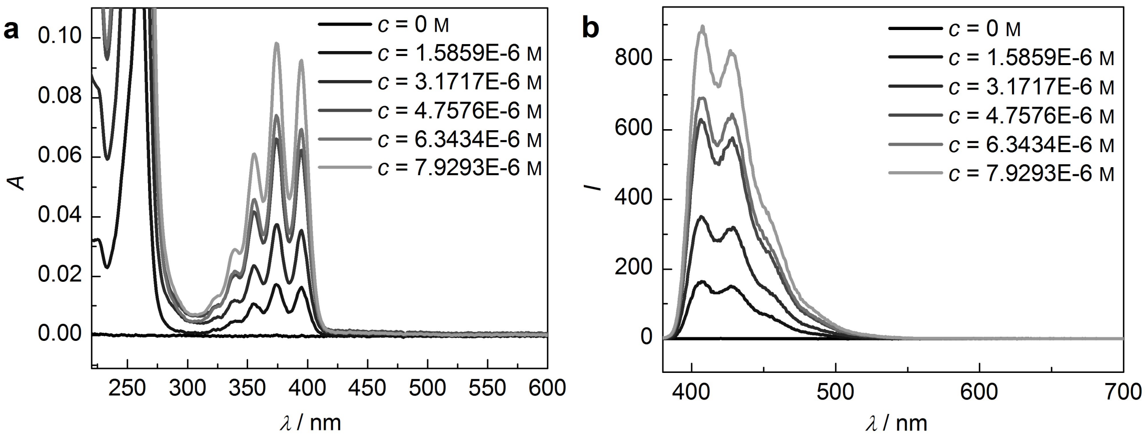

Figure S3. a) Absorption and b) emission spectra of 9,10-diphenylanthracene at different concentrations in cyclohexane used as reference for the determination of fluorescence quantum yields.

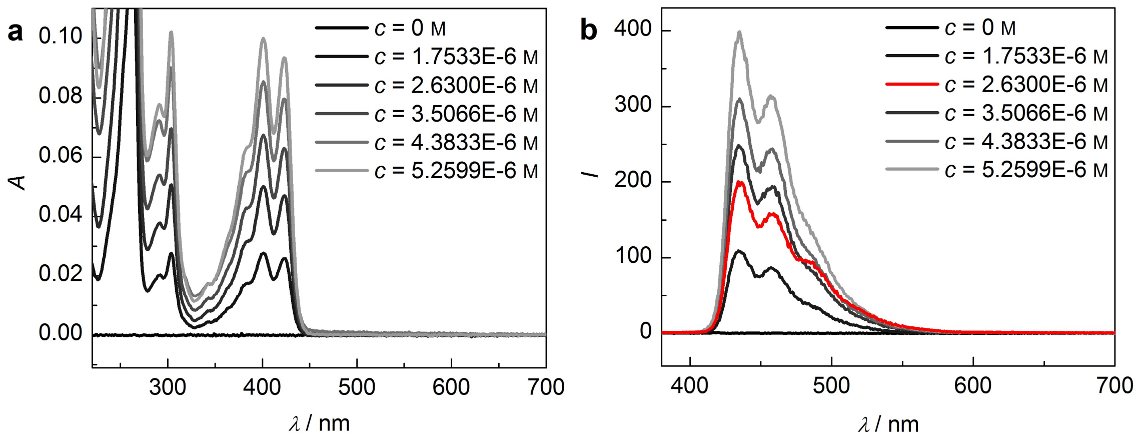

Figure S4. a) Absorption and b) emission spectra of reference compound **14** at different concentrations in acetonitrile used as reference for the determination of fluorescence quantum yields. The spectrum marked red was discarded because of sample contamination.

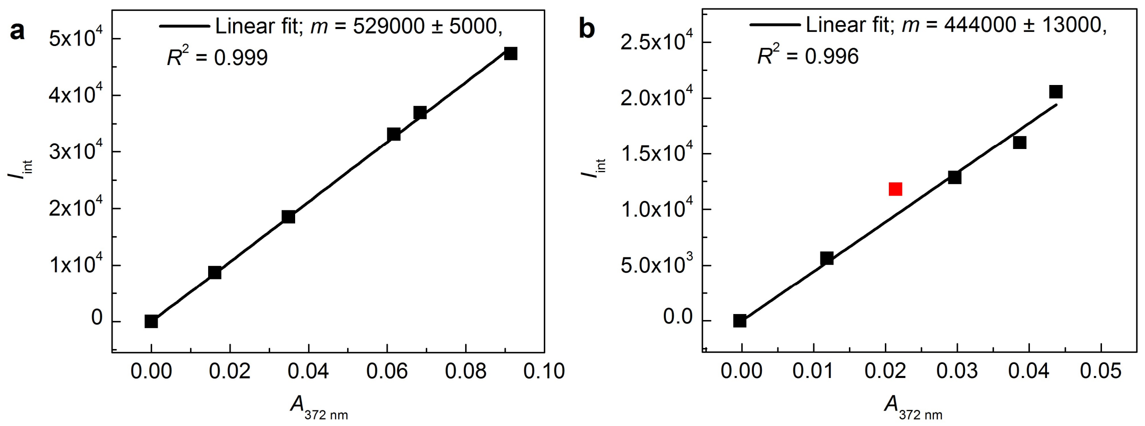

Figure S5. Plots of A vs. I_{int} including linear regression resulting in slope m for a) DPA and b) reference compound **14**. The data point marked red was discarded because of sample contamination.

4. Mechanical Scission Experiments

4.1. General Procedure for Sonication Experiments

A 13 mm probe was used at a frequency of 20 kHz, at 30% of the maximum amplitude of 125 µm. Samples were withdrawn at the beginning, during, and at the end of each experiment and analyzed by GPC. The polymer was dissolved in toluene, placed in a cooled glass vessel (2 °C), and a constant flow of CH_4 was applied to the setup. Then, the mixture was exposed to constant sonication for a given time and samples for GPC and for UV/vis spectroscopy were taken during the process. Removal of the solvent *in vacuo* yielded the cleaved polymer which was used for further ^{1}H-NMR spectroscopy studies.

4.2. Scission Kinetics of PMA-1

Scission rate constants of **PMA-1** were determined by assuming a unimolecular scission process following rate equation

$$\frac{dc_{\text{pol}}}{dt} = -k \cdot c_{\text{pol}}$$

where c_{pol} is the concentration of the initial polymer. Integration gives the integrated rate law of a first-order reaction

$$\ln(c_{\text{pol}}) = -kt + \ln(c_{\text{pol}}^{0})$$

where scission rate constant k can be extracted as the slope of a linear relationship.

The concentration of the polymer during the course of the sonication experiment was either determined by normalized peak-height analyses of the small molecular weight fragment similar to Florea and coworkers[6] assuming that $c_{\text{pol}} \propto h$ where h is the normalized height of the peak in the RI trace of the GPC chromatogram or by UV/vis spectroscopy according to the Beer-Lambert law

$$c_{\text{pol}} = c_{\text{pol}}^{0} - c_{\text{cleaved}} = c_{\text{pol}}^{0} - \frac{A_{400\text{nm}}}{\varepsilon_{400\text{nm}}^{\text{ref}} d}$$

where c_{pol}^{0} is the initial concentration of the polymer at t = 0 irradiation time, $A_{400\ \mathrm{nm}}$ is the absorbance of the polymer sample withdrawn during the irradiation process at 400 nm, d is the optical path length, and $\varepsilon_{400\mathrm{nm}}^{\mathrm{ref}}$ is the known molar absorptivity of reference compound **14** at 400 nm. The linear regressions obtained *via* this method are compiled in Figure S6.

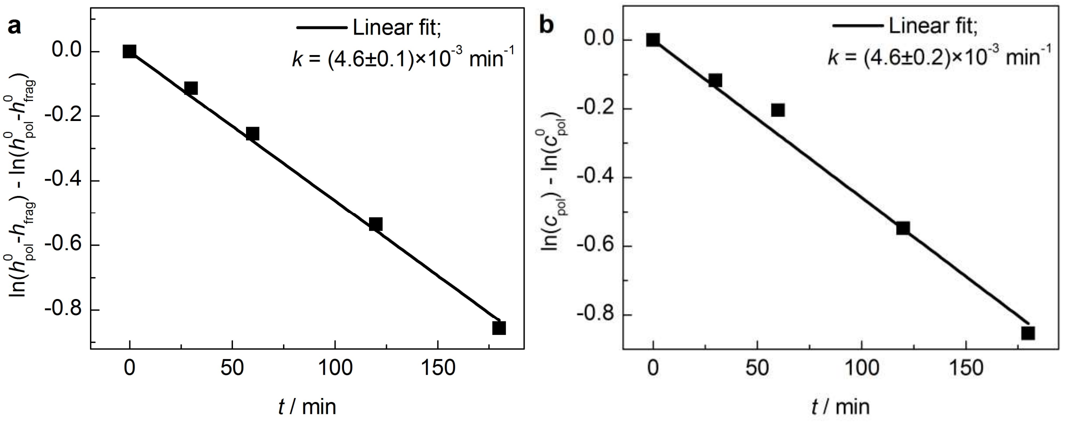

Figure S6. Plots of coefficients determined by a) normalized peak heights of GPC RI chromatograms or b) concentrations calculated from UV/vis absorption spectroscopy of samples withdrawn during the sonication process of **PMA-1** in dependence of irradiation time.

4.3. General Procedure for Compression Experiments

Samples of 6×6 mm were cut out from the respective ca. 0.5 mm thick PHMA film. Compression with a pellet press exerting a weight equivalent of 10 t on the sample was performed for 30 s. The, compressed as well as the uncompressed samples were fixated between two glass slides and analyzed by solid state fluorescence spectroscopy employing an according sample holder.

5. ^{1}H-, ^{13}C-NMR, and HPLC-MS data

5.1. 9-(4-Hydroxyphenyl)ethynylanthracene *N*-(2-hydroxyethyl)maleimide 9,10-Diels-Alder adduct di(α-bromoisobutyrate) 7

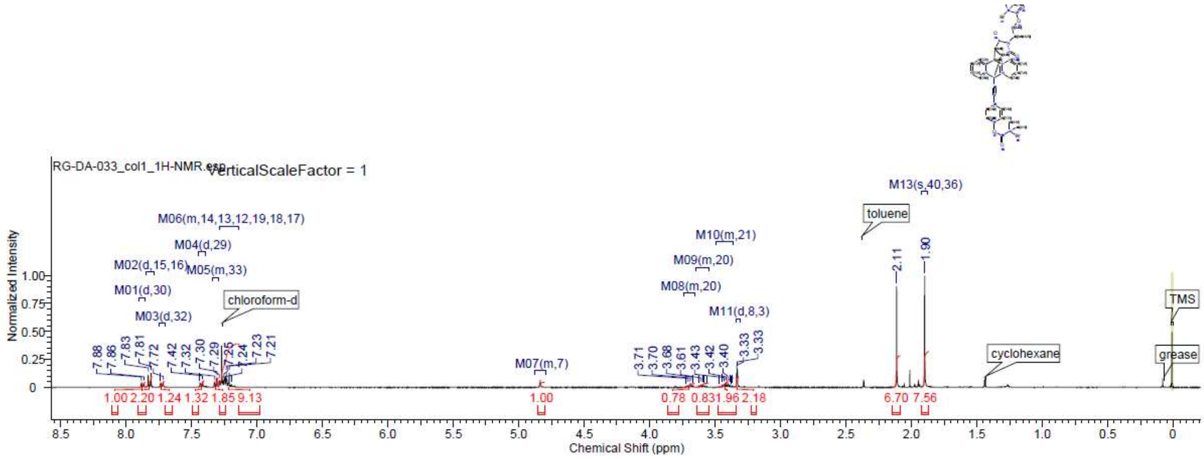

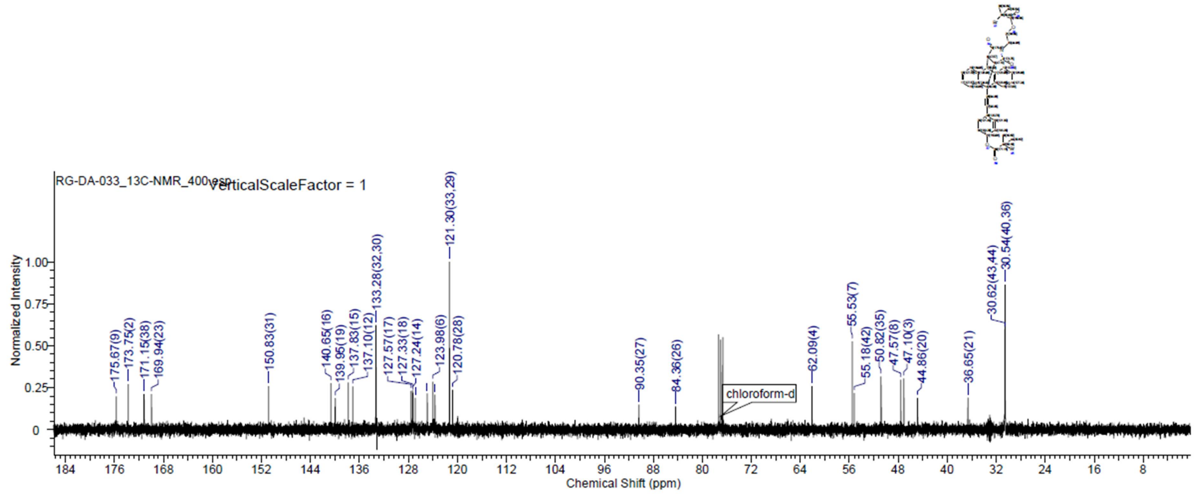

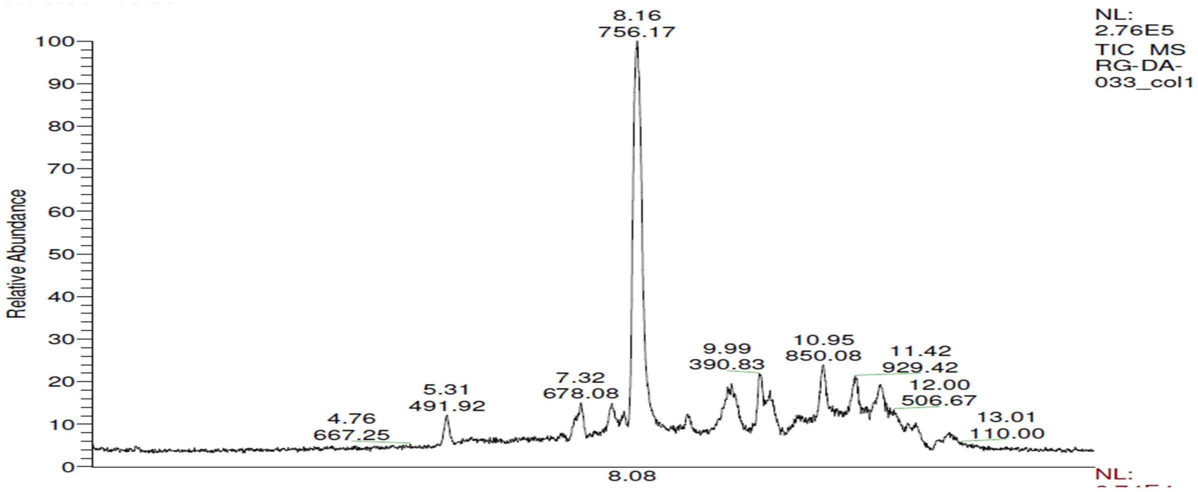

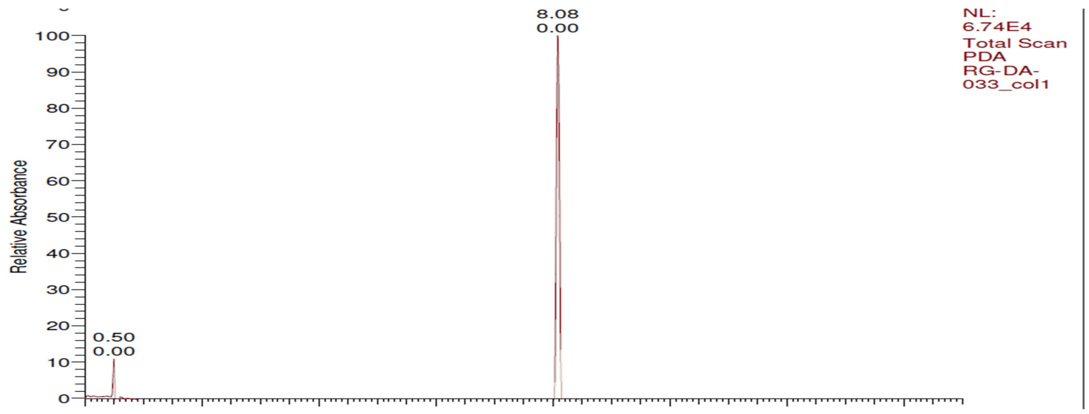

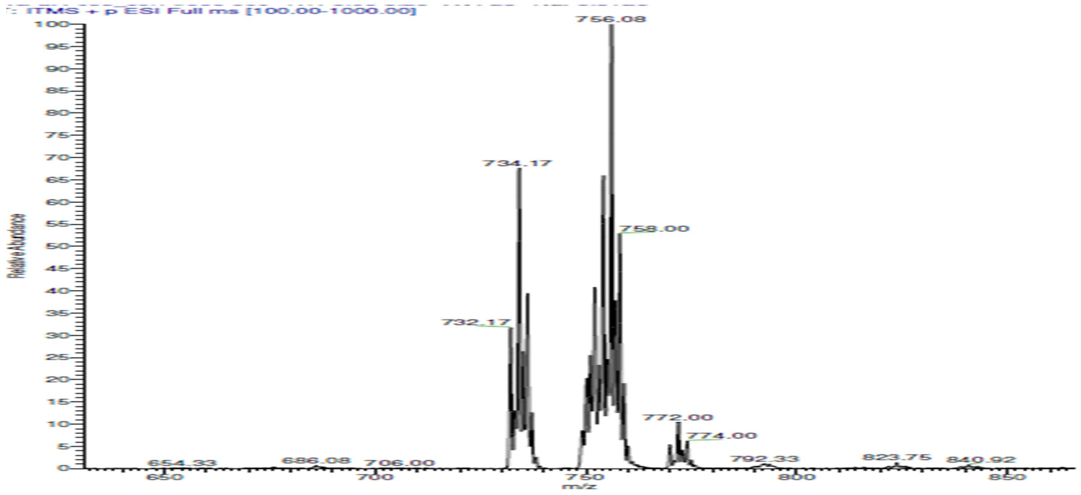

5.2. 9-(4-Hydroxyphenyl)ethynylanthracene *N*-(2-hydroxyethyl)maleimide 9,10-Diels-Alder adduct dimethacrylate 8

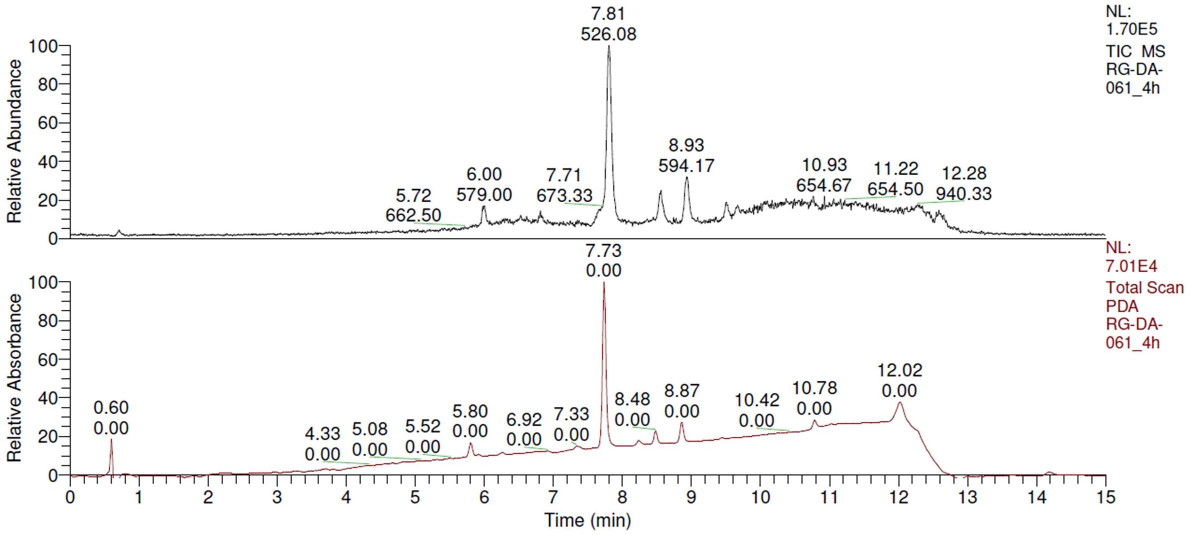

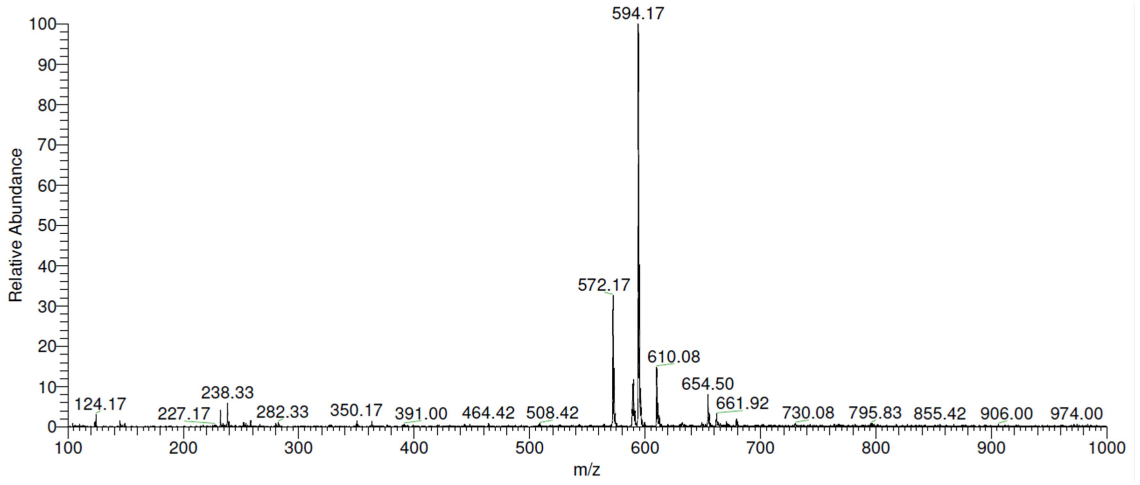

5.3. 9,10-Di(4-anisylethynyl)anthracene *N*-(2-hydroxyethyl)maleimide 1,4-Diels-Alder adduct tri(α-bromoisobutyrate) 13

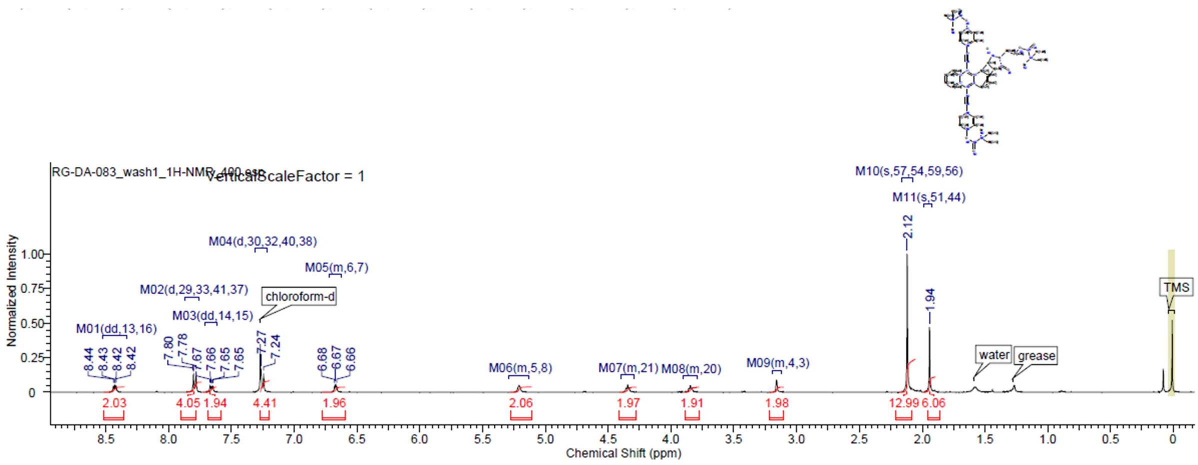

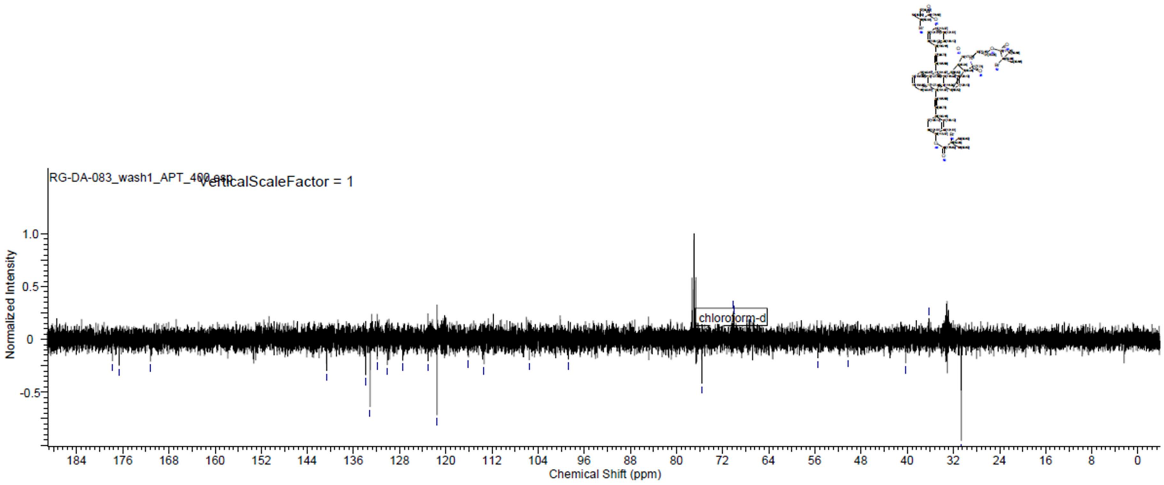

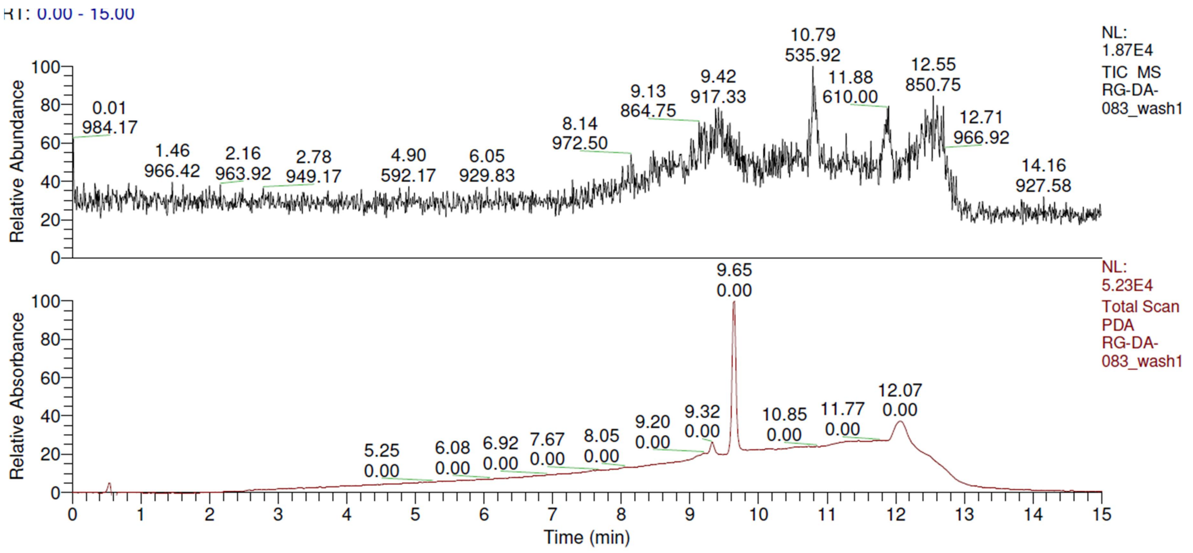

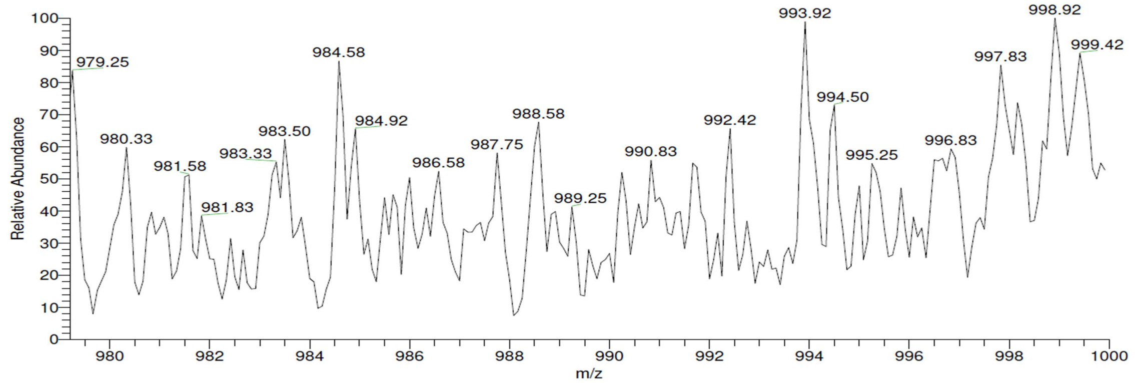

5.4. 9-((4-Anisyl)ethynyl)anthracene 14

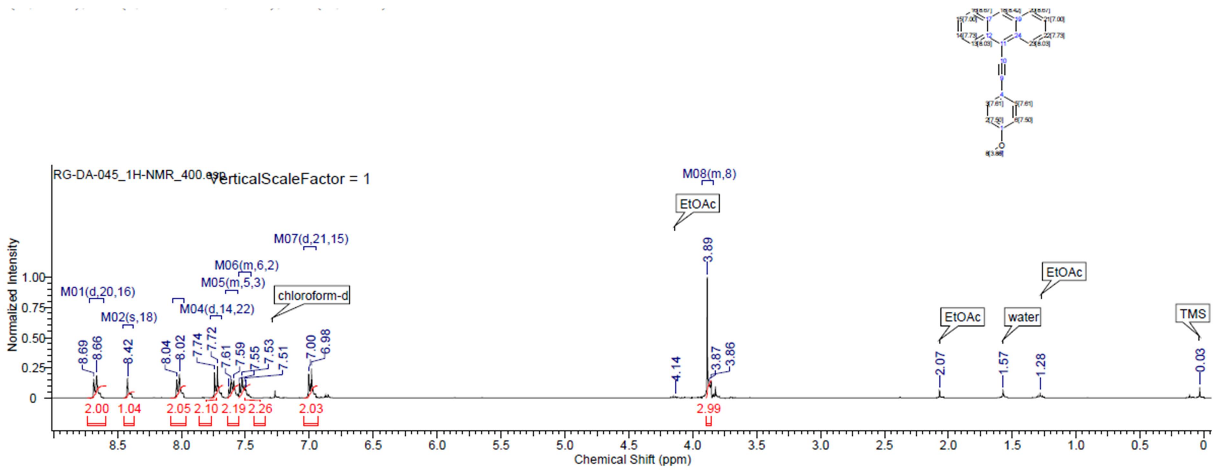

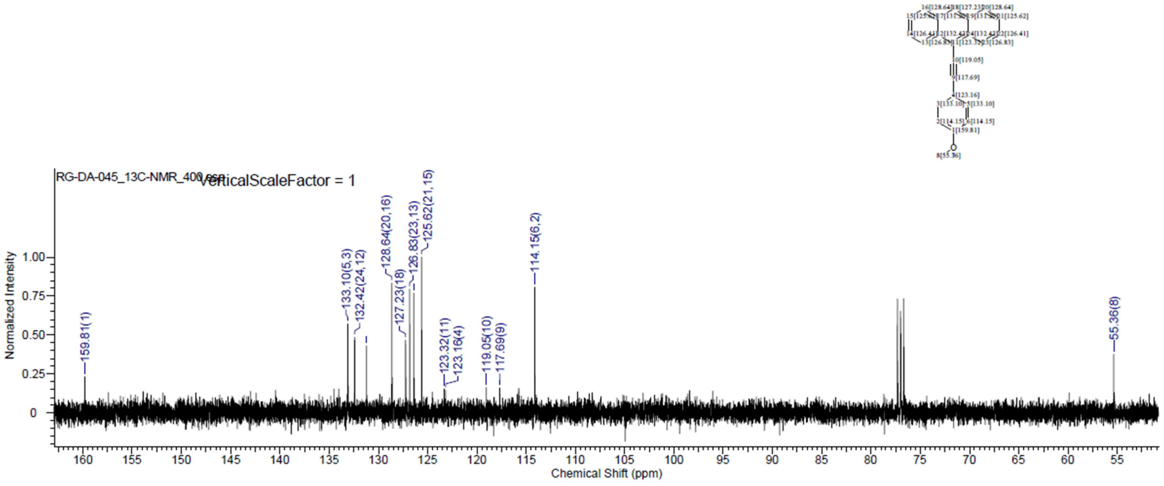
RG-DA-045_13C-NMR_400.esp
VerticalScaleFactor = 1
Normalized Intensity
159.81(1)
133.10(5,3)
132.42(24,12)
128.64(20,16)
127.23(18)
126.83(23,13)
125.62(21,15)
123.32(11)
123.16(4)
119.05(10)
117.69(9)
114.15(6,2)
55.36(8)
Chemical Shift (ppm)

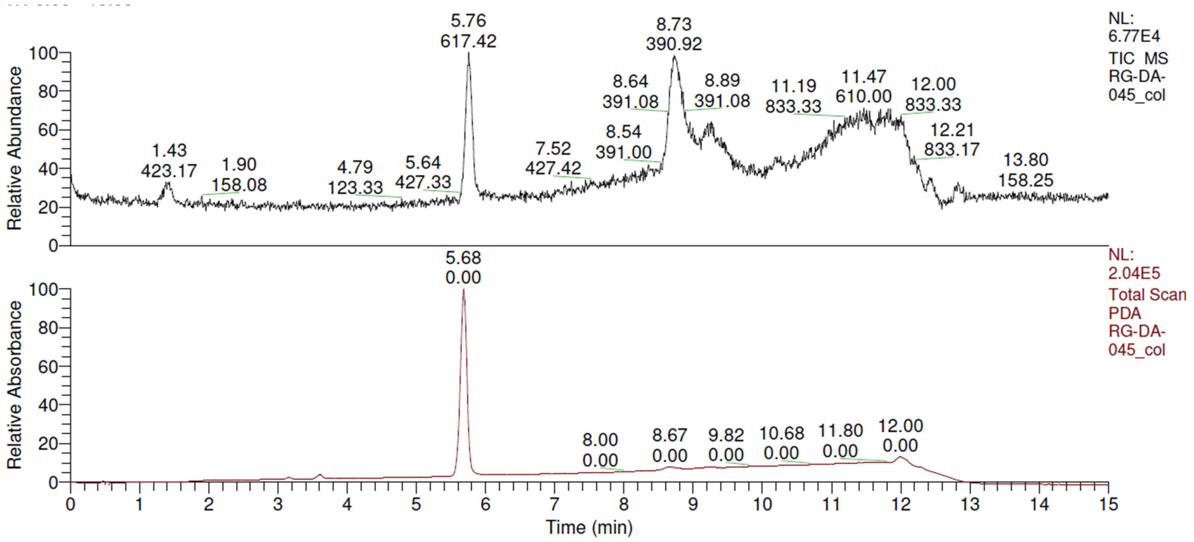
NL:
6.77E4
TIC MS
RG-DA-
045_col
Relative Abundance
5.76
617.42
8.73
390.92
1.43
423.17
1.90
158.08
4.79
123.33
5.64
427.33
7.52
427.42
8.54
391.00
8.64
391.08
8.89
391.08
11.19
833.33
11.47
610.00
12.00
833.33
12.21
833.17
13.80
158.25
NL:
2.04E5
Total Scan
PDA
RG-DA-
045_col
Relative Absorbance
5.68
0.00
8.00
0.00
8.67
0.00
9.82
0.00
10.68
0.00
11.80
0.00
12.00
0.00
Time (min)

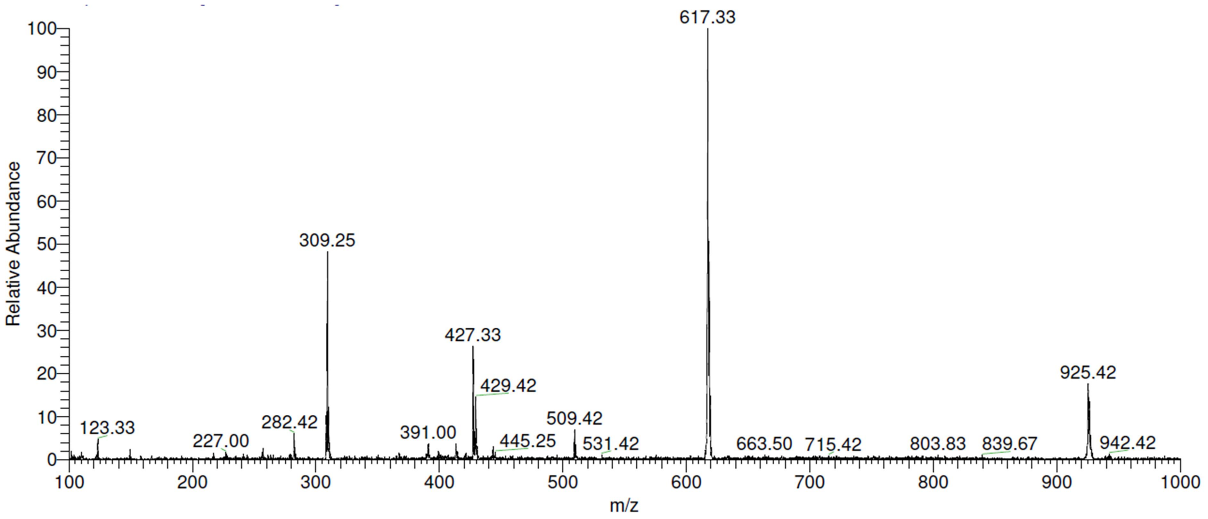
Relative Abundance
617.33
309.25
427.33
429.42
925.42
123.33
227.00
282.42
391.00
445.25
509.42
531.42
663.50
715.42
803.83
839.67
942.42
m/z

5.5. 9,10-Bis-((4-anisyl)ethynyl)anthracene *N*-methylmaleimide 1,4-Diels-Alder adduct 15

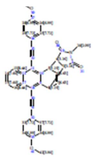

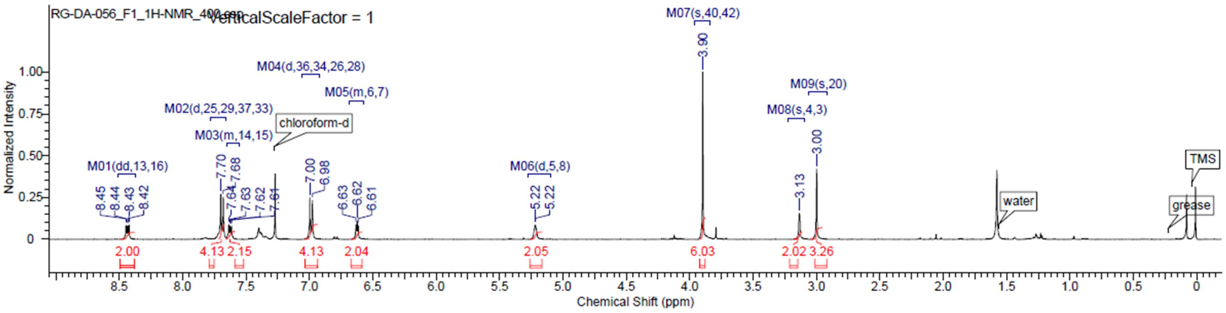

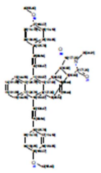

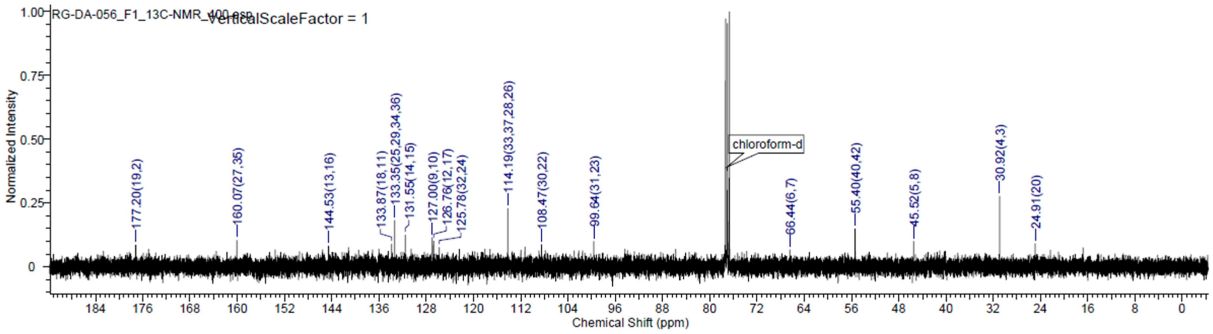

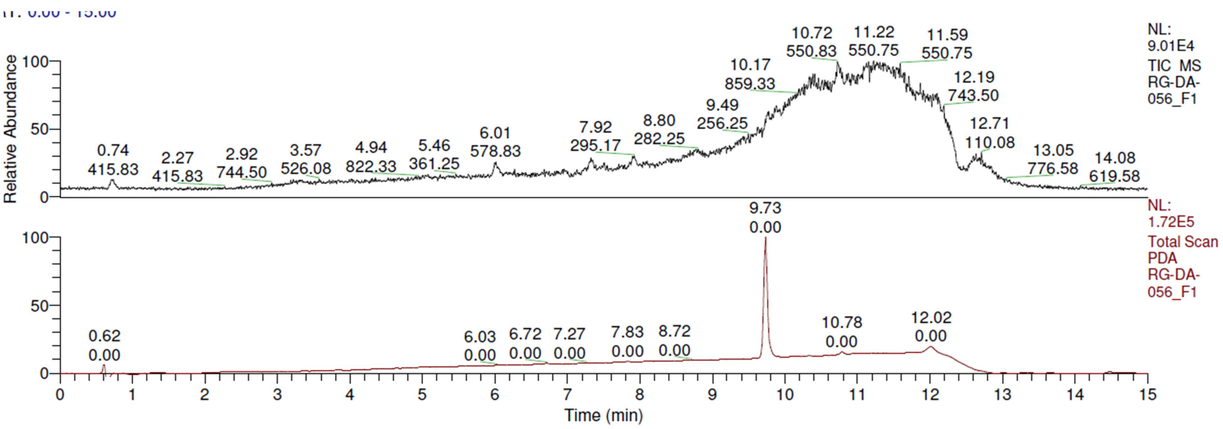

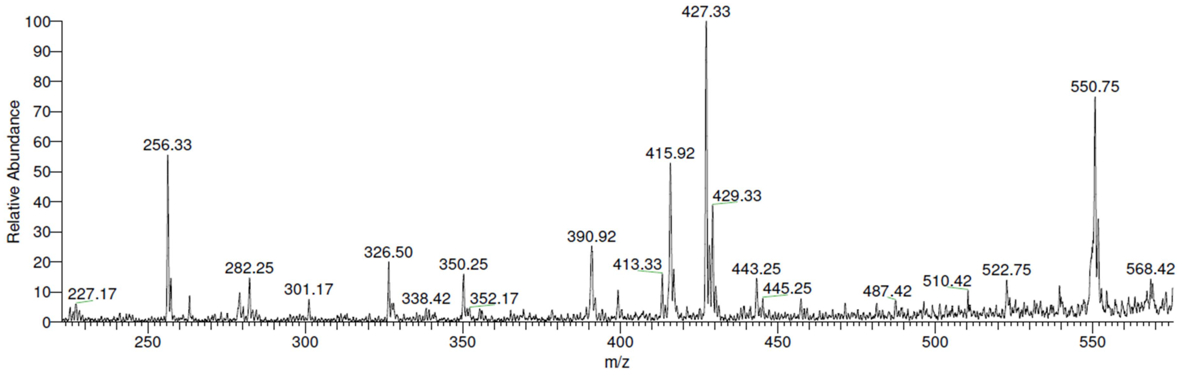

5.6. 9,10-Bis-((4-anisyl)ethynyl)anthracene 16

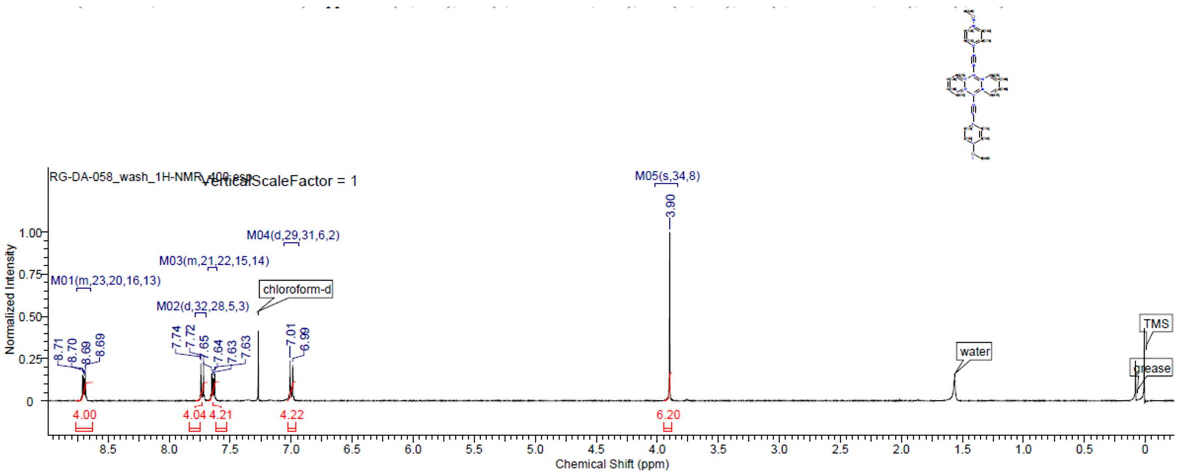

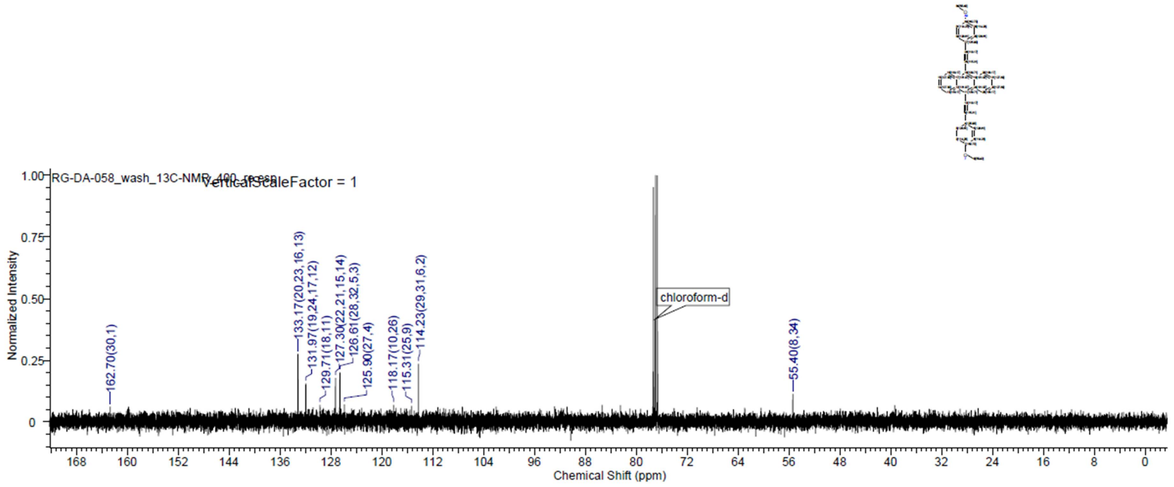

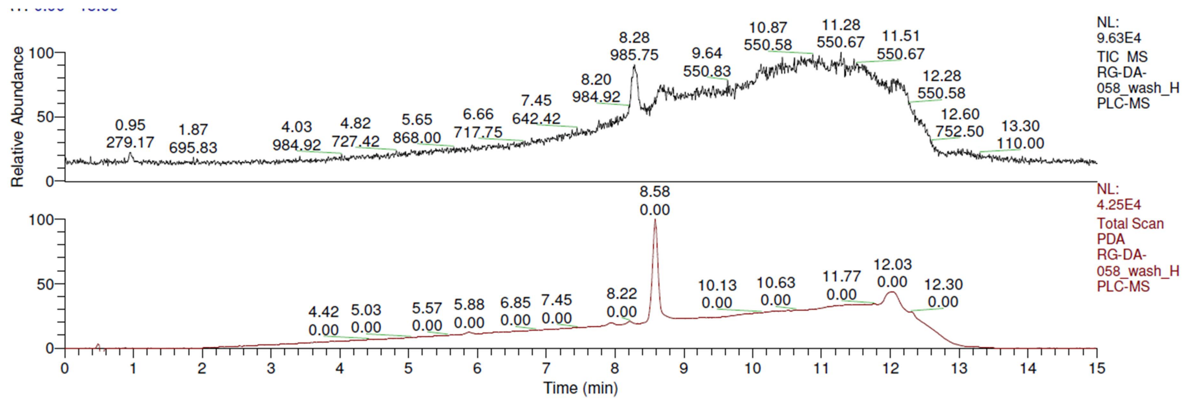

NL: 9.63E4
TIC MS RG-DA-058_wash_HPLC-MS
NL: 4.25E4
Total Scan PDA RG-DA-058_wash_HPLC-MS
Relative Abundance
Time (min)
8.28 985.75
9.64 550.83
10.87 550.58
11.28 550.67
11.51 550.67
12.28 550.58
12.60 752.50
13.30 110.00
8.20 984.92
7.45 642.42
6.66 717.75
5.65 868.00
4.82 727.42
4.03 984.92
1.87 695.83
0.95 279.17
8.58 0.00
12.03 0.00
12.30 0.00
11.77 0.00
10.63 0.00
10.13 0.00
8.22 0.00
7.45 0.00
6.85 0.00
5.88 0.00
5.57 0.00
5.03 0.00
4.42 0.00

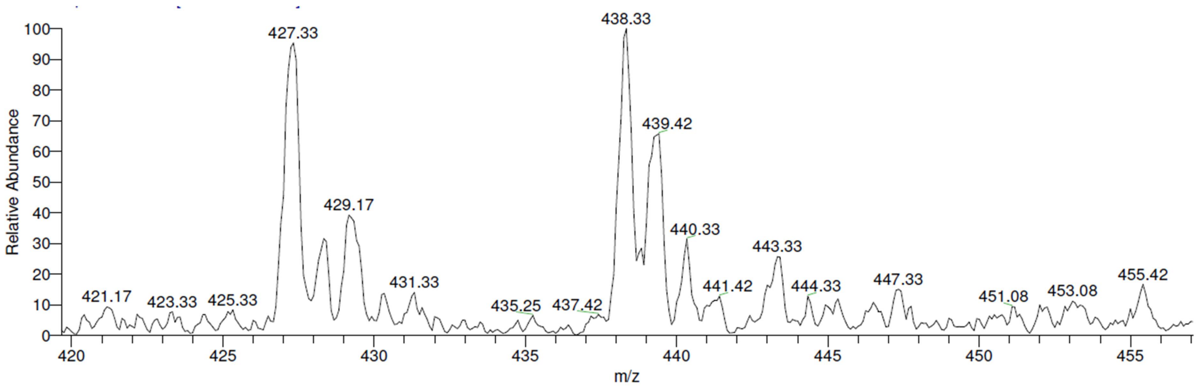

Relative Abundance
m/z
427.33
429.17
431.33
438.33
439.42
440.33
441.42
443.33
444.33
447.33
451.08
453.08
455.42
421.17
423.33
425.33
435.25
437.42

6. References

1 G. R. Fulmer, A. J. M. Miller, N. H. Sherden, H. E. Gottlieb, A. Nudelman, B. M. Stoltz, J. E. Bercaw and K. I. Goldberg, *Organometallics*, 2010, **29**, 2176–2179.

2 J. A. Syrett, G. Mantovani, W. R. S. Barton, D. Price and D. M. Haddleton, *Polym. Chem.*, 2010, **1**, 102.

3 U. Resch-Genger and K. Rurack, *Pure Appl. Chem.*, 2013, **85**, 2005–2013.

4 A. M. Brouwer, *Pure Appl. Chem.*, 2011, **83**, 2213–2228.

5 K. Suzuki, A. Kobayashi, S. Kaneko, K. Takehira, T. Yoshihara, H. Ishida, Y. Shiina, S. Oishi and S. Tobita, *Phys. Chem. Chem. Phys.*, 2009, **11**, 9850–9860.

6 M. Florea, *J. Appl. Polym. Sci.*, 1993, **50**, 2039–2045.
